# Bioinspired synthesis of ultra-small copper-aspartate BioMOF nanodots using sodium caseinate for targeted curcumin delivery

**DOI:** 10.1038/s41598-025-18880-4

**Published:** 2025-10-08

**Authors:** Reyhane Rezaee, Maryam Tohidi, Banafsheh Rastegari, Sedigheh Zeinali

**Affiliations:** 1 Department of Nanochemical Engineering, Faculty of Advanced Technologies, Shiraz University, Shiraz, 71946-84334 Iran; 2 Diagnostic Laboratory Sciences and Technology Research Center, School of Paramedical Sciences, Shiraz University of Medical Sciences, Shiraz, 71837-53335 Iran

**Keywords:** Copper-aspartate, Biological metal organic framework, Ultrasmall nanodots, Sodium caseinate, Curcumin, Folic acid, Biomimetic synthesis, Nanoparticle synthesis, Targeted therapies

## Abstract

In this study, an attempt was made to create of copper-aspartate (Cu-Asp) biological metal organic framework (bioMOF), inspired by the process of bio-mineralization. Ultra-small nanodots of biocompatible Cu-Asp was synthesized as a carrier for curcumin (CCM) in the presence of sodium caseinate (CAS) in an aqueous medium at room temperature. Synthesis was performed in the presence of different amounts of CCM (10, 15, and 20 mg). Two different morphologies of the nanorod and nanodots (for the first time) were obtained. To target the drug delivery processes, the folic acid (FA) factor was used during the synthesis of ultra-samll nanodots. X-ray diffraction, scanning and field emission scanning (SEM and FESEM) and transmission electron microscopies, Fourier transform infrared (FT-IR), UV–Vis, and Thermogravimetric (TG) analyses were performed to characterize the synthesized nanostructures. The drug loading efficiency and capacity percentages for CAS/CCM/FA@Cu-Asp (5.1 and 74%, respectively) indicated that the encapsulation of the drug was appropriate. In addition, the rate of drug release at different pH values was investigated, and the results showed different performances of the nanocarriers at pH = 5.5, 6.8, and 7.4. The release of CCM from CAS/CCM@Cu-Asp was 60% at pH = 5.5 after 3 days. At pH 7.4, only 10% CCM was released after 4 h and remained nearly stable. The potential cytotoxicity was evaluated using the MTT assay in Normal skin fibroblast and HeLa (positive folate receptor cancer cell) cell lines. The highest SI was observed for CAS/CCM/FA@Cu-Asp (2.15).

**Supplementary Information:**

The online version contains supplementary material available at 10.1038/s41598-025-18880-4.

## Introduction

Metal–organic frameworks (MOFs), also known as coordination polymers, are crystalline materials formed through self-assembly of metal ions and organic ligands via strong coordination bonds. MOFs exhibit remarkable properties, including customizable topologies, high porosity, low density, and excellent thermal and chemical stability. Their well-ordered porous polycrystalline structures offer numerous chemically reactive sites, such as metal centers and functional groups on ligands, making them versatile for applications like gas storage, catalysis, and separation. The structural tunability of MOFs allows for tailored designs, establishing them as a cornerstone in materials science^1,2^.

Biological metal–organic frameworks (BioMOFs) are a novel class of porous materials designed for biomedical applications^3,4^. They incorporate nontoxic metal ions, such as Ca^2^⁺, Mg^2^⁺, Fe^2^⁺, Cu^2^⁺, and Zn^2^⁺, and biocompatible ligands, including endogenous biomolecules or exogenous compounds. The endogenous ligands like amino acids, peptides, nucleobases, and saccharides^5^, sourced from renewable and biodegradable materials, provide multiple coordination sites and diverse functional groups, enabling control over interactions, reaction sites, and structural flexibility^6^. This results in biocompatible composites ideal for therapeutic and drug delivery applications^7,8^*.* Examples include ZIF-8^9,10^, MIL-100(Fe)^11,12^, HKUST^13^, MOF-199^14^, MIL-101(Fe)–NH_2_^15^_,_ β-cyclodextrin MOF^16^ and NH_2_ -functionalized MIL-53(Fe)^17^ for delivering anticancer drugs such as CCM, DOX and 5-fluorouracil. BioMOFs’ compatibility with biological environments enhances their potential for seamless integration into physiological systems^18,19^. Amino acid-based BioMOFs were constructed employing amino acids such as L-Glutamic acid, aspartic acid^20^, glycine and lysine^21^. Thses BioMOFs leverage the dual functionality of amino acids, which contain carboxyl (–COOH) and amino (–NH_2_) groups, serving as ideal ligands^22^.

Copper-based MOFs, utilizing Cu^2^⁺ ions and ligands such as 1,4-benzenedicarboxylic acid (terephthalic acid)^23^, 2-aminoterephthalic acid^24^, 1,3,5-benzenetricarboxylic acid^25^ L- or D-Aspartic acid^26^, are promising for drug delivery. These nanoscale architectures excel in drug encapsulation, controlled release, and targeted delivery^27,28^ particularly for chemotherapeutic agents, showing potential in cancer treatment due to their ability to release drugs selectively at tumor sites^29,30^.

Ultra-small nanobioMOFs, typically less than 10 nm, are advanced drug delivery platforms. Their nanoscale size improves cellular uptake, tissue penetration, and targeted drug delivery, combining MOFs’ tunable porosity and stability with biocompatibility and biodegradability. These properties make nanobioMOFs highly effective for delivering therapeutic agents with precision, particularly in biomedical contexts requiring minimal invasiveness and high specificity^31,32^.

Biomolecules can be integrated into MOFs in two ways. Post-synthesis incorporation places biomolecules within pre-formed MOFs, constrained by pore size. Alternatively, biomineralization-inspired synthesis incorporates biomacromolecules—like proteins, enzymes, DNA, or polysaccharides—during MOF formation, requiring mild conditions, such as low temperatures and biocompatible solvents, to preserve biomolecule integrity^33^. The latter accelerates MOF formation, enhances structural diversity, and improves biomolecule stability under physiological conditions, benefiting applications like targeted drug delivery and tissue engineering. Biomolecules in the reaction medium promote diverse morphologies and efficient release into physiological environments, triggered by stimuli like pH variations^34^.

Curcumin (CCM), a hydrophobic polyphenol from turmeric, offers antioxidant, anti-inflammatory, antimicrobial, and anticancer properties but suffers from low bioavailability and solubility^35,36^. MOF-based delivery systems address these limitations^37^. Bonding CCM’s keto/enol moieties to metal ions like Zn^2^⁺ or Cu^2^⁺ enhances hydrolytic stability. Protein-based nanocarriers, such as sodium caseinate (CAS), derived from milk casein, improve CCM’s dispersibility and bioactivity due to their amphiphilic properties. NaCAS nanoparticles are effective for poorly soluble drugs, enhancing permeability and retention effects^38,39^. Recent literature highlights MOFs’ role in CCM delivery, offering methods to protect and deliver water-insoluble drugs to target sites^40–42^.

Stimulus-responsive drug delivery systems enhance MOF-based therapies by responding to triggers like pH, temperature, enzymes, or folate receptors^43^. In cancer tissues, which are more acidic than healthy tissues, pH-responsive MOFs increase drug release^33^. Folic acid (FA) targets folate receptors, overexpressed in cancers like breast, lung, and colon, enabling precise drug delivery to tumor cells^45–47^. This improves therapeutic efficacy while minimizing off-target effects.

This study develops ultra-small, tumor-targeting nanobioMOF drug carriers based on Cu^2+^ and aspartate (Cu-Asp) frameworks, incorporating FA as a targeting agent and CAS/CCM. Synthesized at room temperature via biomineralization, the process uses an alkaline medium with an aspartate linker to dissolve FA, yielding CAS/CCM/FA@Cu-Asp nanocarriers. Characterization employed scanning electron microscopy (SEM), transmission electron microscopy (TEM), X-ray diffraction (XRD), Fourier transform infrared spectroscopy (FTIR), UV–Vis spectroscopy, and thermogravimetric analysis (TGA). Cytotoxicity was assessed using the MTT assay on normal skin fibroblast (NSF) and HeLa (folate receptor-positive) cell lines, demonstrating the carriers’ potential for targeted cancer therapy. These nanobioMOFs combine biocompatibility, targeted delivery, and stimulus-responsive properties, positioning them as promising tools for precision medicine.

## Experimental section

### Chemicals

Copper Nitrate (Cu(NO_3_)_2_.3H_2_O, 98%), Sodium hydroxide (NaOH, 98%), aspartic acid (C_4_H_7_NO_4,_ 98%), and folic acid (C₁₉H₁₉N₇O₆, FA, 98%) were purchased from MERCK. Phosphate-buffered saline (PBS) and Dulbecco’s modified Eagle medium (DMEM) were purchased from Sigma-Aldrich. Sodium Caseinate (CAS, 98%) was supplied by the Karen Company. CCM (95%) was obtained from JinTai Biological Company (China). The materials for the MTT assay were purchased from Sigma-Aldrich. All chemicals were used without further purification. Distilled water was used in all experiments. Hela, A549, and normal human fibroblast cell lines were purchased from the Pasteur Institute of Iran.

## Methods

The one-pot synthesis of Cu-Asp as a nanocarrier was performed in the presence of CAS, CCM, and FA at room temperature.

### Synthesis of Cu-Asp in the absence and presence of CAS, CAS/CCM, and CAS/CCM/FA at room temperature

The synthesis of the Cu-Asp bioMOF has been previously reported^26^. Initially, 1.0 mmol (240 mg) of hydrated copper (II) nitrate was added to 15.0 ml of distilled water. Further, 0.8 mmol (107 mg) of aspartic acid was dissolved in 2.0 ml of a NaOH solution (1.0 M). Subsequently, aspartate solution was added to the Cu^2+^ solution while stirring. The synthesis of Cu-Asp occurred immediately. The final blue product was washed three times with distilled water and centrifuged for 10 min at 4000 rpm. Finally, it was dried at ambient temperature. The synthesis was conducted at room temperature. This product was named Cu-Asp.

Cu-Asp was synthesized in the presence of CAS at room temperature as follows (Fig. S1). First, CAS (200.0 mg) was dispersed in distilled water (10.0 ml). The resulting solution was centrifuged to remove the suspended particles and impurities (Fig. S1a). Afterward, 2.0 ml of the alkaline aspartate solution (0.8 mmol (107 mg) Asp, 1.0 M NaOH) and 5.0 ml of aqueous Cu^2+^ solution (1.0 mmol, 240 mg) were added to the CAS solution (Fig. S1b and S1c). The resulting mixture was then stirred for 15 min. The obtained blue product was washed 3 times with water and centrifuged at 4000 rpm for 10 min. (Fig. S1d). The final prototype is referred to as the CAS@Cu-Asp framework (Fig. S1e).

To form a carrier containing CCM, CAS@Cu-Asp was synthesized in the presence of CCM (Fig. S2). First, (10, 15, or 20 mg) mg of CCM was dissolved in 4.0 ml of ethanol. This solution was added to 10.0 ml of CAS solution (200 mg), which had already been centrifuged (Fig. S2a). Then, 2.0 ml of alkaline aspartate solution (0.8 mmol (107 mg) Asp, 1.0 M NaOH) (Fig. S2b) and 1.0 ml of Cu^2+^ solution (1.0 mmol, 240 mg) were added to the CAS/CCM mixture, respectively (Fig. S2c). The color of the mixture changed from dark red to green with the addition of Cu^2+^. The reaction medium was completely mixed using a homogenizer for 2 min (Fig. S2d). After washing 3 times with water and centrifugation for 10 min at 6000 rpm, the final green product was dried at room temperature (Fig. S2e). The final sample was CAS/CCM (10, 15, or 20 mg)@ Cu–Asp. As a control, CCM@Cu-Asp was synthesized using a similar method in the absence of CAS.

The synthesis of the targeted carrier with FA was performed using a similar method, except that 10.0 mg of FA was added to 2.0 ml of an alkaline solution of aspartate (0.8 mmol, 107 mg). The resulting solution was added to 14.0 ml of the prepared CAS/CCM mixture (20. mg CCM in 4.0 ml of ethanol). Afterward, 1.0 ml of an aqueous solution containing Cu^2+^ (1.0 mmol, 240 mg) was added. The reaction medium was mixed with a homogenizer for 2 min. The obtained product was washed with water and centrifuged for 10 min at 5000 rpm. The product was dried at ambient temperature. The final sample was labeled with CAS/CCM (20 mg)/FA@Cu-Asp.

### Characterization techniques

FESEM images were obtained using a Hitachi S-4160 at an accelerating voltage of 20 kV. SEM was performed using TESKAN-Vega 3. The TEM images were acquired by ZEISS-EM10C-100 kV (Germany). The particle size distribution histogram was obtained from TEM images using Image J software and plotted using OriginPro software. XRD patterns were obtained using a D8 ADVANCE (Bruker, Germany) with Cu-Kα radiation (λ = 0.1542 nm). The XRD patterns were obtained using an angular step of 0.02° and sampling time of 1 s per step in the 2θ range of [5–50°]. Absorbance measurements were performed using a Shimadzu 1601 PC UV–Vis spectrophotometer. FTIR spectra were obtained using a tensor II (BRUKER Germany). TGA was performed using a TGA/DSC 1 − Thermogravimetric Analyzer (Mettler-Toledo International Inc.) under a nitrogen atmosphere from room temperature to 800 °C at a heating rate of 5 °C min^−1^. The absorbance of the solutions was recorded using a Polar Star Omega plate reader (BMG LABTECH, Germany).

### Evaluation of cytotoxicity with MTT assay

Cytotoxicity was evaluated using fibroblasts (normal cells) and HeLa (folate receptor-positive) cell lines. For this purpose, each cell line was subcultured in complete medium containing high-glucose DMEM supplemented with 10% heat-inactivated fetal bovine serum in the presence of 1% penicillin/streptomycin at 37.0 °C under a humidified atmosphere of 5% CO_2_. After reaching 90% confluence, the cells were detached using 0.25% prewarmed trypsin. Then, 1.0 × 10^4^ cells/well of HeLa and A549 cells and 1.5 × 10^4^ cells/well of normal skin fibroblasts/well were seeded into 96-well plates overnight under the above conditions. The next day, the medium was replaced with 100 μL of fresh culture medium containing 5.0, 10.0, 20.0, 30.0, 50.0, 75.0, and 100.0 μg mL^−1^ of different types of samples with CCM treatment at final concentrations of 280.0, 140.0, 70.0, 35.0, 17.5, 8.7, and 4.4 μg mL^−1^ was also performed using 1% ethanol as the negative control. After 24, 48, and 72 h of incubation, cells were washed twice with PBS and incubated in fresh medium containing 0.5 mg mL^−1^ of MTT solution. The plates were covered with aluminum foil and incubated in an incubator under CO_2_ for 4 h. The culture medium was then removed, and the formazan crystals were dissolved in 100.0 μL of 100% DMSO. The absorbance of the solution was measured at wavelengths of 570 and 630 nm and correlated with the formazan optimum absorbance and background turbidity using a plate reader (Tecan infinite-200 M Pro, Tecan Co, Switzerland), respectively. The cell viability was determined as follows:

$$\text{Cell Viability }\left(\text{\%}\right)= \left(\frac{\text{A Sample }-\text{ A Blank}}{\text{A Negative Control }-\text{ A Blank}}\right)\times 100$$

### Evaluation of the CCM loading capacity and loading efficiency

In general, 2.0 mg of the total precipitated sample was dissolved in 50.0 µl of 3.0 M HCl, which was diluted with ethanol to 2.0 ml, and then its Uv–Vis spectrum was recorded, and the absorbance was obtained at a wavelength of 425 nm. Accordingly, the CCM concentration was determined using the calibration curve equation (Fig. S3). Based on the following two Eqs. (1 and 2), the percentage of the loaded drug capacity (DLC) and loaded drug efficiency (DLE) were calculated:

1 $${\text{DLC}}\left( \% \right) \, = \, \left( {\text{amount of loaded drug}} \right)/\left( {\text{amount of drug loaded NPs}} \right) \, \times {1}00$$

2 $${\text{DLE}}\left( \% \right) \, = \, \left( {\text{amount of loaded drug}} \right)/\left( {\text{total amount of feeding drug}} \right) \, \times { 1}00$$

### Evaluation of the CCM release rates and mechanisms

To evaluate the release of CCM from CAS/CCM@Cu-Asp, phosphate buffer solution (PBS) was used as the release medium at pH 5.5, 6.8, or 7.4 (cancer tissue and blood simulation). Briefly, 5.0 mg of CAS/CCM@Cu-Asp was poured into a glass test tube containing a PBS solution (10.0 ml). The PBS solution contained 20 w/w% Tween20 and 0.1 mg/ml BSA. For the degradation of the nanocarrier and CCM release, the test tubes were maintained at a constant temperature by rotary rotation. 500.0 µl of the solution was removed using a micropipette and centrifuged at specified time intervals (15 min, 30 min, 1 h, 2 h, 3 h, 4 h, 24 h, 48 h, and 72 h). The supernatant was replaced with fresh stock solution (PBS buffer, BSA, and Tween20). After sampling from the drug release medium at different pH values, each aliquot was mixed in a 1:1 volume ratio with ethanol (50:50 v/v) prior to UV–Vis spectrophotometric measurement, in order to ensure complete solubilization of CCM. Calibration curves at each pH were also prepared using the same 50:50 ethanol-buffer mixture to maintain consistency in solvent environment (Fig. S3). Finally, the percentage of released CCM at pH 5.5, 6.8, or 7.4 was investigated by calculating its concentration from its maximum absorbance peak at 425 nm and the calibration curve equation in each pH, as well as the total amount of CCM loaded in the nanocarrier. Kinetic release data are presented as the average ± SD (n = 3) in three independent experiments.

Drug release profiles of CCM from CAS/CCM@Cu-Asp nanocarrier were fitted to four classical kinetic models: Zero-order, First-order, Higuchi, and Korsmeyer–Peppas. The experimental data at pH 7.4, 6.5, and 5.5 were analyzed using nonlinear regression with Python 3.11 (SciPy/NumPy libraries).

For each pH condition, model parameters and correlation coefficients (R^2^) were calculated, and the best-fitting model was selected based on goodness-of-fit. Model fitting for Zero-order and First-order models was performed over the experimental time ranges before level off. The Higuchi and Korsmeyer–Peppas model were fitted within the initial release phase (~ 60% cumulative release, Mt/M∞ ≤ 0.6).

## Result and discussion

### Investigation of the effect of presence of CAS on the synthesis of Cu-Asp and CCM@Cu-Asp frameworks

**Fig. 1:**
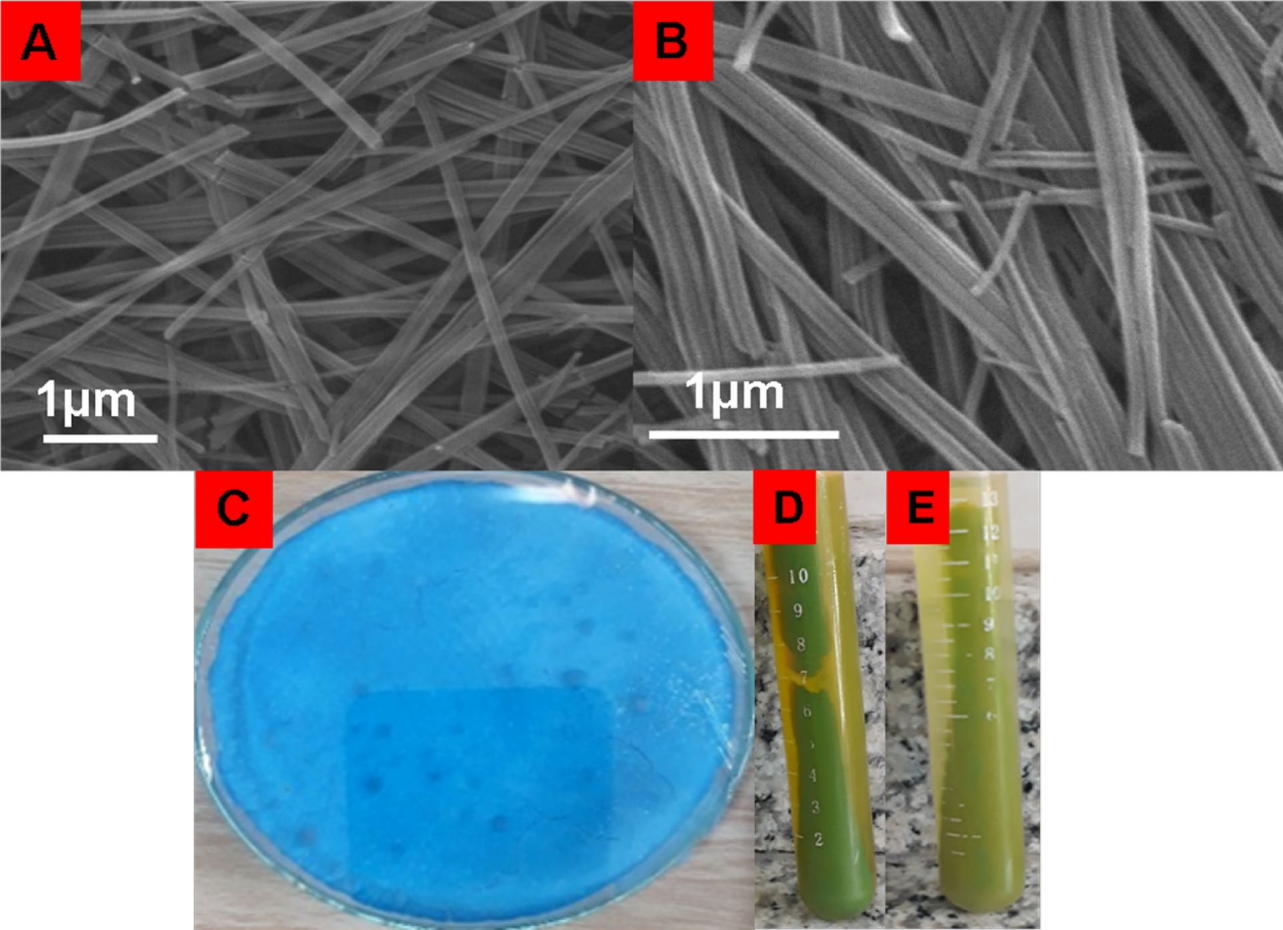
SEM images of (**A**) Cu-Asp and (**B**) CAS@Cu-Asp and (**C**) photograph of Cu-Asp, (**D**) CCM@Cu-Asp and (**E**) CAS/CCM@Cu-Asp after washing and centrifugation.

SEM analysis was performed to investigate the morphologies of the synthesized structures (Figs. 1A,B). The Cu-Asp bioMOF was previously synthesized^26^. The synthesized nanostructure was blue in color and had a rod-shaped morphology (Fig. 1A,C). This bioMOF was used as the CCM carrier. The color of the Cu-Asp carrier containing CCM was green, owing to the presence of the drug (CCM@Cu-Asp). However, Cu-Asp alone was not able to efficiently retain CCM in its construct; hence, the major part of the drug leaked during the rinsing process, which can be distinguished by the color of the supernatant (Fig. 1D). On the other hand, based on the results of the MTT test, this framework was not sufficiently biocompatible for use as a carrier because of the presence of Cu^2+^ in the structure. To enhance biocompatibility, the Cu-Asp bioMOF was synthesized in the presence of CAS biomolecules at room temperature (CAS@Cu-Asp). The SEM image of CAS@Cu-Asp is shown in Fig. 1B. In the presence of CAS, the morphology was also rod-shaped (Fig. 1B). The CAS@Cu-Asp retained CCM in its porosity during the washing process (Fig. 1E). As reported in the literature, the bioactivity and dispersibility of CCM are enhanced in the presence of CAS protein^48^. Additionally, CAS can exhibit superior interactions with CCM compared with other biomolecules, including whey, soy proteins, and gum arabic. Possible interactions between CCM and CAS occur because of the presence of hydrophobic regions in both molecules. CCM contains a lipophilic tail that allows it to bind to the hydrophobic regions of proteins, such as CAS. This binding may occur through noncovalent interactions such as hydrophobic and van der Waals forces^49^. Therefore, CAS@Cu-Asp was selected as a suitable CCM carrier.

### Investigation of the effect of different CCM concentrations on the CAS@Cu-Asp morphology

**Fig. 2:**
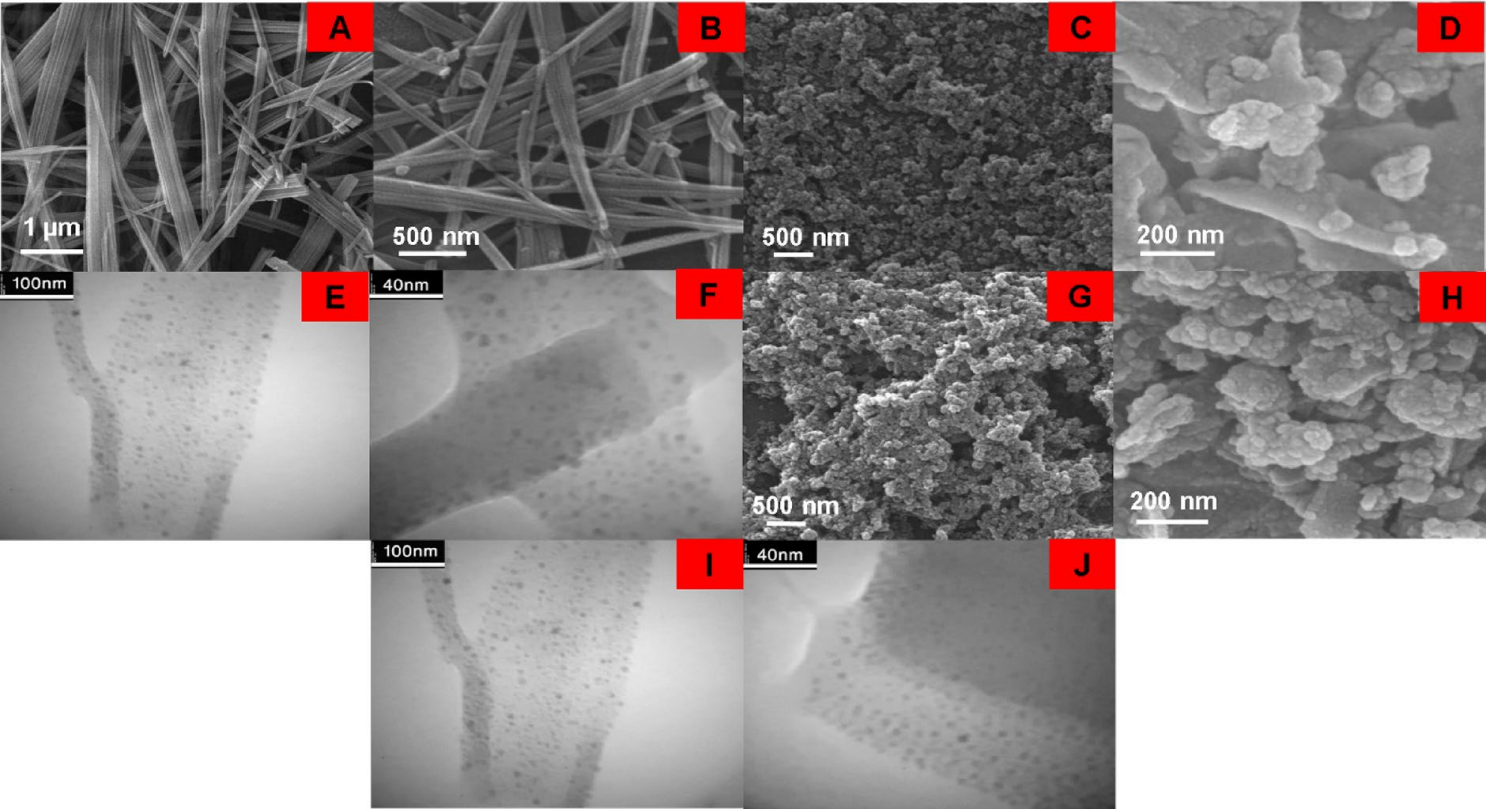
SEM images of CAS/CCM)10 mg(Cu-Asp (**A**) and CAS/CCM)15 mg(Cu-Asp (**B**); SEM (**C**), FESEM (**D**), and TEM (**E**–**F**) images of CAS/CCM(20 mg)@Cu-Asp; SEM (**G**), FESEM (**H**), and TEM (**I**–**J**) images of CAS/CCM(20 mg)/FA@Cu-Asp.

SEM, FESEM, and TEM analyses were used to investigate the effect of different amounts of CCM on the morphology and size of CAS@Cu-Asp. Figure 2 shows the SEM images of the synthesized frameworks containing the CAS@Cu-Asp, CAS/CCM(10 mg)@Cu-Asp, CAS/CCM(15 mg)@Cu-Asp, and CAS/CCM(20 mg)@Cu-Asp. For CAS/CCM(20 mg)@Cu-Asp, FESEM and TEM images were also obtained (Fig. 2). Cu-Asp, CAS@Cu-Asp, and CAS/CCM(10 mg)@Cu-Asp exhibited rod-shaped morphologies with average diameters of 104.70, 59.43, and 53.25 nm, respectively (Figs. 1A-B, 2A, and S4 A-C). In the case of CAS/CCM(15 mg)@Cu-Asp, beside nanorods (with average diameter of 10.46 nm and shorter length), some spherical nanoparticles (with average diameter of 6.3) were obtained (Fig. 2B). The nanorod diameters decreased with a narrower size distribution owing to the presence of more CCM in the synthesis medium (Fig. S4 A-D). In the case of CAS/CCM(20 mg)@Cu-Asp, the morphology changed completely from nanorods to nanodots. (Fig. 2C–F). The naodots average particle size was estimated to be 5.39 nm with a narrow size distribution based on the histogram obtained from the TEM images (Fig. S4E). The change in morphology from nanorod to nanodots with increasing the amount of CCM suggests that a different growth mechanism occurs when a higher amount of CCM is present in the reaction medium. CCM can bind to CAS, as reported in the literature. The CAS/CCM complex can form through noncovalent interactions such as hydrophobic and van der Waals forces^49^. This complex can indeed serves as a template for the biomineralization processes^49^. The CAS/CCM complex can interact with Cu^2+^ ions. The presence of two phenolic and two ketonic groups in CCM provides multiple binding sites for metal ions. This CAS/CCM/Cu^2+^ can act as a template for Cu-Asp framework deposition and is responsible for the biomineralization process. This phenomenon can direct and control the formation of MOFs^50^. The morphology and size alteration can also be explained by the limited availability of the framework materials for growth due to the increase in the amount of template. Overall, the results highlight the role of the template in modifying the morphology and size of the framework material. Understanding these effects is crucial for designing and controlling the fabrication of nanomaterials with the desired morphologies and properties.

As mentioned earlier, in drug delivery processes, small spherical nanomaterials exhibit the highest cellular uptake in certain cells^51^. Therefore, among the different products, an ultra-small CAS/CCM(20 mg)@Cu-Asp nanodot was chosen.

### Investigation of the effect of FA on the synthesis of CAS/CCM@Cu-asp

FA was used to target CAS/CCM(20 mg)@Cu-Asp to FA overexpressed cancer cells (CAS/CCM(20 mg)/FA@Cu-Asp). To dissolve FA, an alkaline medium or organic solvent is required. Herein, FA was dissolved in an alkaline medium of aspartate solution without using any organic solvent. Figure 2G–J shows the SEM, FESEM, and TEM images of the CAS/CCM(20 mg)/FA@Cu-Asp. Ultra-small nanodots with an average particle size of 5.14 nm and a narrow size distribution were obtained, as confirmed by the size distribution histogram (Fig. S4F). Moreover, FA did not have a noticeable effect on the size and morphology of the nanobioMOF.

### Investigation of the crystallinity of CAS@Cu-Asp bioMOF in the presence of CCM and FA by XRD analysis

**Fig. 3:**
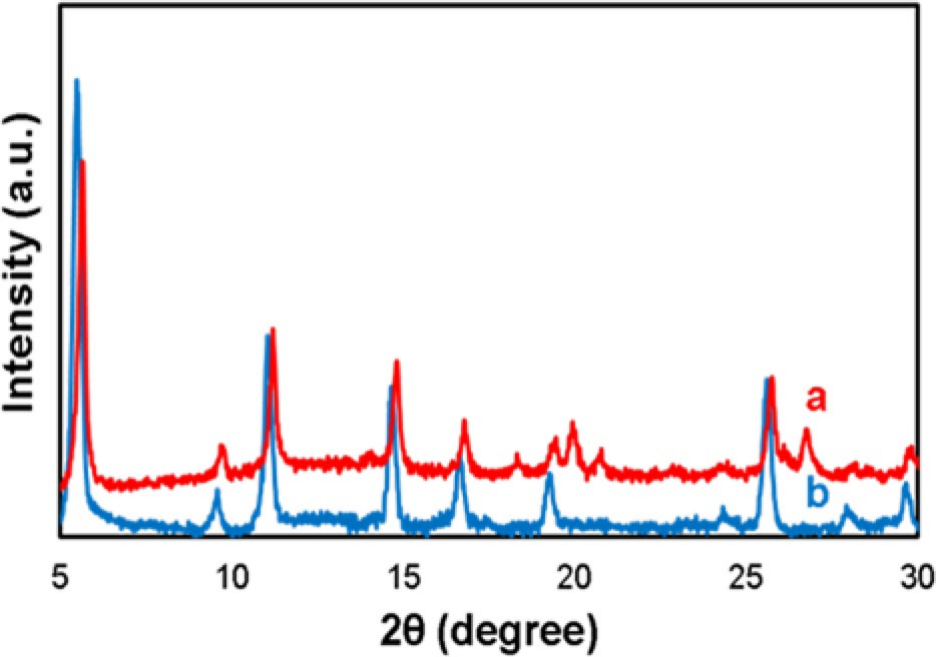
XRD spectra of (**a**) CAS/CCM (20mg)/FA@Cu-Asp/FA and (**b**) Cu-Asp.

XRD analysis was conducted to evaluate the crystal structure of CAS/CCM (20 mg)/FA@Cu-Asp (nanodots) compared to Cu-Asp (rod morphology) (Fig. 3). The XRD patterns revealed that the crystallinity of CAS/CCM(20 mg)/FA@Cu-Asp is largely consistent with that of the Cu-Asp framework (Fig. 3a,b)^22,27^. However, distinct alterations were observed, including changes in peak intensity ratios, slight peak shifts (e.g., ~ 0.2–0.5° shift in the peaks), and the emergence of new peaks at 2θ ≈ 20, 20.76 and 26.72° in CAS/CCM (20 mg)/FA@Cu-Asp. These changes are attributed to the morphological transition from rod-shaped Cu-Asp to nanodot-shaped CAS/CCM (20 mg)/FA@Cu-Asp and the incorporation of biomolecules (CAS, CCM, and FA).

The reduction in particle size to the nanoscale and the shape transformation induce lattice strain and alter atomic arrangements, leading to peak broadening, shifts, new peak formation and different peaks relative intensities^52–54^. Additionally, the presence of biomolecules likely introduces local structural distortions through coordination with Cu^2^⁺ or hydrogen bonding, further modifying peak intensities and positions^55,56^. For instance, similar XRD peak shifts and new peaks have been reported in biomolecule-incorporated MOFs, where guest molecules disrupt lattice periodicity^55,56^. These structural modifications provide critical insights into the interactions between the Cu-Asp bioMOF and biomolecules, reflecting changes in the local crystal environment^57^.

### Investigation of the presence of biomolecules in the Cu-Asp framework by FTIR analysis

**Fig. 4:**
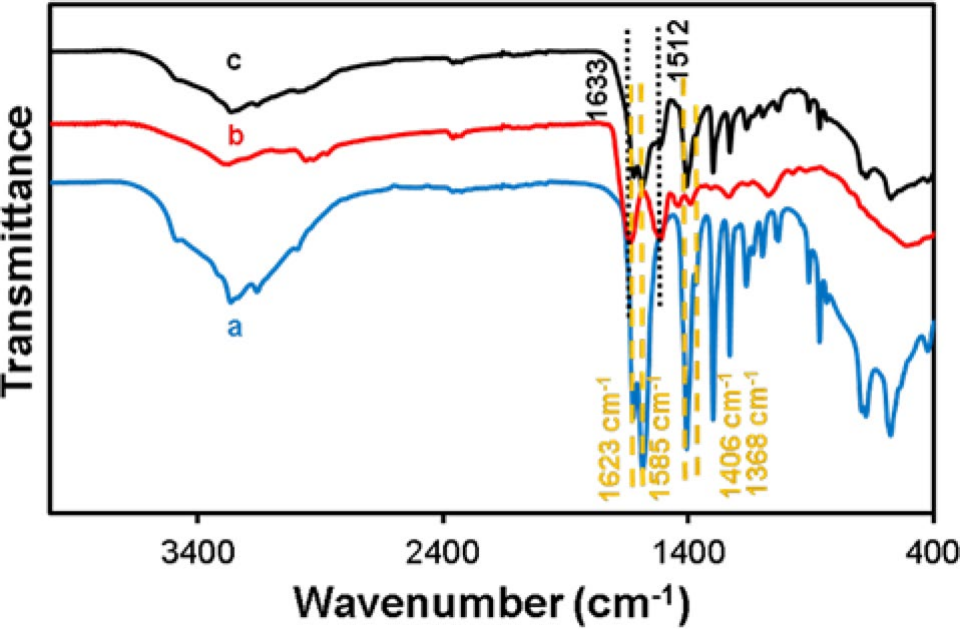
FTIR spectra of (**a**) Cu-Asp, (**b**) CAS, and (**c**) Cas@Cu-Asp.

FTIR spectroscopy was employed to identify functional groups in CAS, Cu-Asp, and CAS@Cu-Asp. (Fig. 4). For Cu-Asp bioMOF (Fig. 4a), broad peaks at 1623/1585 cm^−1^ and 1406/1368 cm^−1^ were observed, corresponding to the asymmetric and symmetric stretching vibration modes of the COO^-^-Cu^2+^ coordination, confirming the formation of metal–ligand bonds^28,58^. These peaks were also present in CAS@Cu-Asp (Fig. 4c), indicating that the Cu-Asp structure is retained in the nanocomposite despite the presence of CAS in the synthesis process. (Fig. 4c). Additionally, two prominent peaks at 1633 cm⁻^1^ and 1512 cm⁻^1^ in CAS@Cu-Asp (Fig. 4b,c) confirm the incorporation of the CAS biomolecule, likely attributed to C=O and C=C stretching modes, respectively, which are characteristic of its chemical structure ^38^.

For bioMOFs containing biomolecules CAS, CCM and FA, peak broadening, shifting, and overlap occur. These effects arise from: hydrogen bonding between functional groups of CAS/CCM/FA (CAS likely contributes C=O and C=C groups, while CCM and FA introduce –OH and -NH groups) and Cu-Asp, altering vibrational energies; coordination interactions of biomolecules with Cu^2^⁺ by donating electron pairs, modifying electron density and causing peak shifts; and conformational heterogeneity within the bioMOF, leading to broadened peaks due to varied molecular environments^59,60^. Overlap occurs as functional groups like C=O and COO⁻ absorb in similar regions (1600–1700 cm⁻^1^), making peaks indistinguishable". These interactions obscure distinct peak resolution, reflecting complex intermolecular dynamics.

### Investigation of the presence of biomolecules in the Cu-Asp framework by UV–Vis spectroscopy

**Fig. 5:**
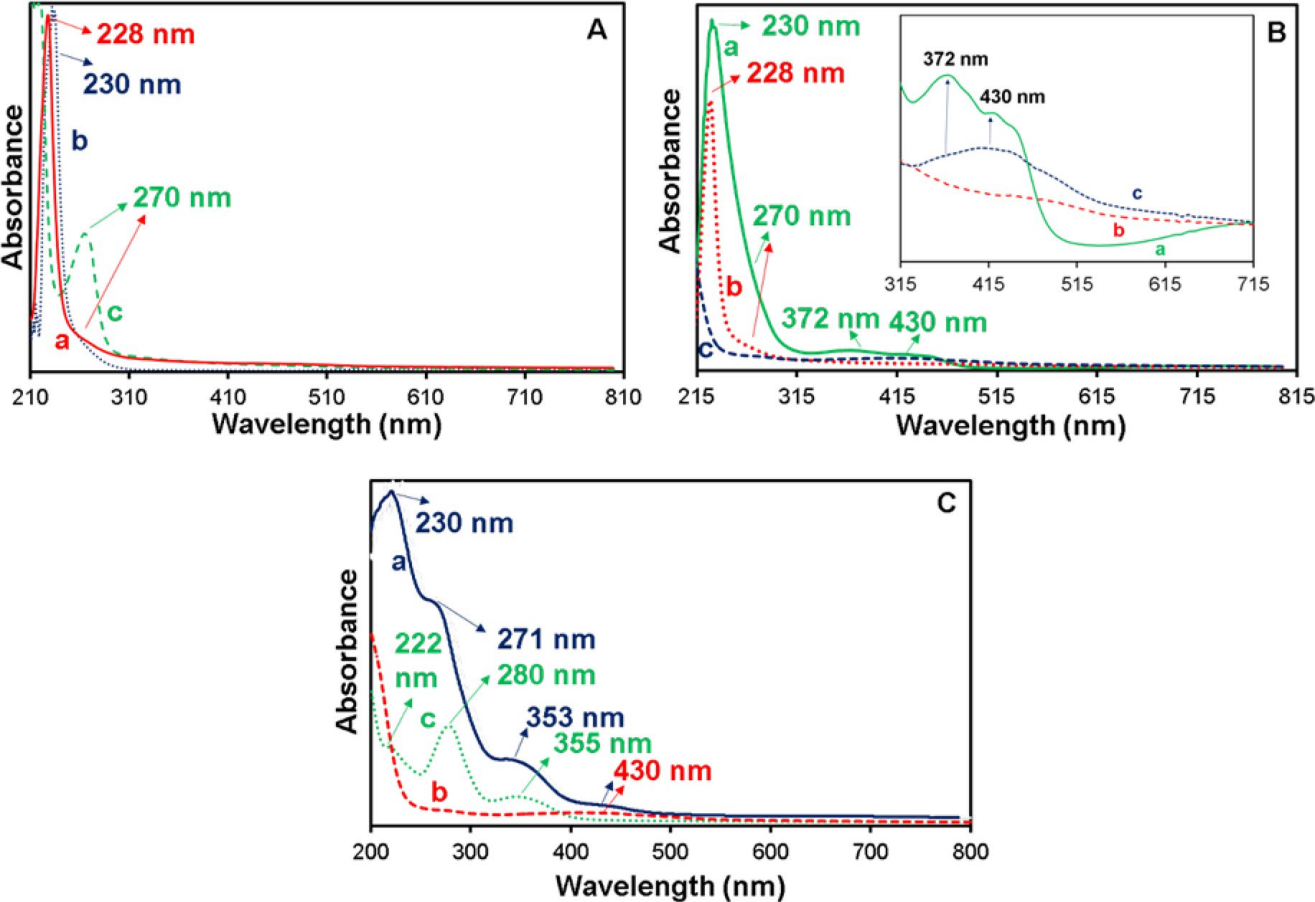
UV − Vis spectra of (**A**) Cu-Asp (a), CAS@Cu-Asp (b), CAS (c), (**B**) CAS/CCM(20 mg)@Cu-Asp (a), CAS@Cu-Asp (b) and CCM (c( and (**C**) CAS/CCM(20 mg)/FA@Cu-Asp (a), CCM (b) and FA (c). Inset in B shows magnified regions to highlight spectral differences.

UV–Vis spectroscopy was used to evaluate the absorption peaks of free CCM, CAS, FA, Cu-Asp, CAS@Cu-Asp, CAS/CCM (20 mg)@Cu-Asp, and CAS/CCM(20 mg)/FA@Cu-Asp. As shown in Fig. 5A (a-b), a sharp peak at 228 nm in the CAS@Cu-Asp spectrum could be also noticed in Cu-Asp with a slight redshift (230 nm). The observed shift can be attributed to the presence of CAS and the corresponding interactions with the Cu-Asp counterparts. CAS has a peak at 270 nm (Fig. 5A c). This peak also indicated in CAS@Cu-Asp very little as a peak shoulder (Fig. 5A b). A comparison of CAS/CCM (20 mg)@Cu-Asp and CAS@Cu-Asp showed a higher CAS load in the framework due to the appearance of a broader shoulder at 270 nm (Fig. 5Ba and b). This observation confirmed the production of the CAS/CCM complex and its role as a template for Cu-Asp biomineralization, which resulted in higher biomolecule loading. In addition, the Cu-Asp peak in the CAS/CCM (20 mg)@Cu-Asp framework exhibited some shift with respect to CAS@Cu-Asp, which could be due to interactions (Fig. 5Ba and b).

The CCM absorption spectrum shows broad peaks between 370 and 430 nm ^61^. Accordingly, to confirm the encapsulation of CCM in the CAS/CCM (20 mg)@Cu-Asp framework, the UV–Vis spectrum of the bioMOF was compared with that of free CCM. The results are shown in Fig. 5B a and c. In the CAS/CCM(20 mg)@Cu-Asp spectrum, two absorption peaks at 372 and 430 nm are observed, which are related to the encapsulated CCM. The observed shift in the CCM absorption peaks can be related to the CCM interaction with the other components in the framework (Fig. 5B).

To confirm the presence of FA in CAS/CCM(20 mg)/FA@Cu-Asp, its UV–Vis spectrum was obtained and compared with that of pure FA. As shown in Fig. 5C a and c, FA exhibited three index absorption peaks at 222, 280, and 355 nm, which were observed in the CAS/CCM(20 mg)/FA@Cu-Asp with some shifts (230, 271, and 353 nm). In this case, the indicated shift was due to FA encapsulation in the framework and the creation of possible interactions. In some cases, peak overlapping was occurred. The peaks at 230 and 271 nm are also related to Cu-Asp, CAS and the peak at 430 nm corresponded to CCM, respectively (Fig. 5C a and b). These results confirm the presence of different biomolecules in the framework.

### Investigation of the presence of biomolecules in the Cu-Asp framework determined by TGA

**Fig. 6:**
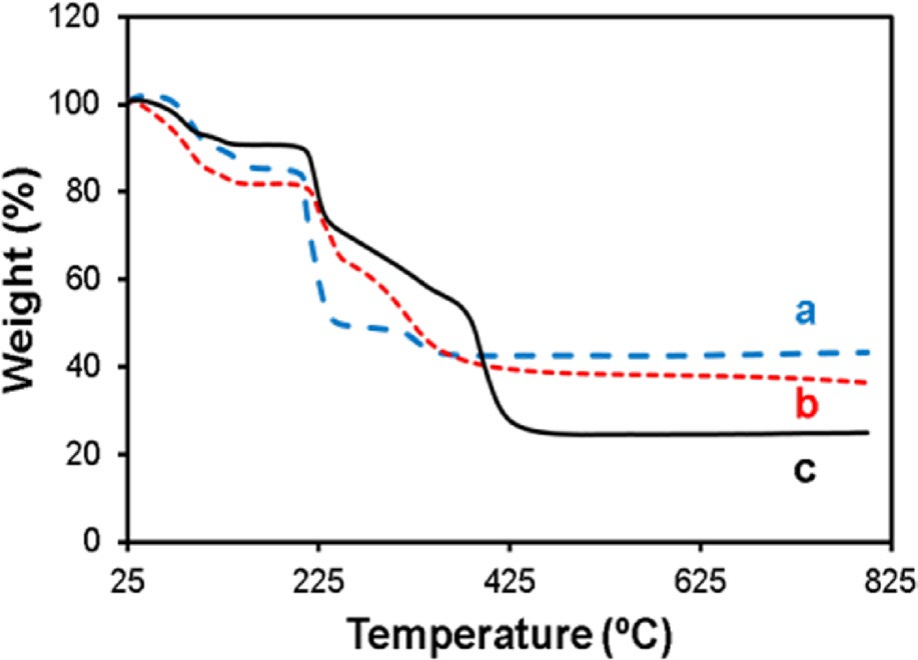
TGA thermograms of (**a**) Cu-CAS, (**b**) CAS@Cu-Asp and (**c**) CAS/CCM (20 mg)/FA@Cu-Asp.

TGA analysis was used to investigate the presence of biomolecules in the Cu-Asp framework. Figure 6 shows the TGA plots of Cu-Asp, CAS@Cu-Asp and CAS/CCM(20 mg)/FA@Cu-Asp. The obtained results indicated the alteration of the TGA curve of Cu-Asp by loading different biomolecules containing CAS, CCM, and FA (Fig. 6). These biomolecules can interact with the framework structure to influence its thermal properties. This can result in various effects on the TGA curve, such as a shift in the decomposition temperature and influence on thermal stability, changes in the peaks or steps observed in the TGA curve, and increased or decreased mass loss. The thermal degradation of L-Asp occurred in three main steps, observed between 210–260 °C, 360–415 °C, and 480–640°C^58^. However, when L-Asp was coordinated with Cu^2+^ to form Cu-Asp bioMOF, its thermal stability decreased. The thermal degradation of u-Asp MOF started at lower temperatures (118–137 °C, 189–198 °C, 289–317 °C) (Fig. 6a and S5A). Nonetheless, the addition of CAS slightly improved the thermal stability of Cu-Asp bioMOF (119–137 °C, 214–238 °C, 290–347 °C) (Fig. 6b and S5B), while the incorporation of CCM and FA leds to a more enhancement in its thermal stability (213–229 °C, 214–238 °C, 379–408 °C) (Fig. 6c and S5C). These shifts in decomposition temperatures and reduced weight losses indicate that biomolecules interact with the Cu-Asp framework through coordination with Cu^2+^ ions or encapsulation, enhancing thermal stability^58^. These findings underscore the potential of biomolecule-incorporated bioMOFs for applications requiring enhanced thermal stability.

### Investigation of CCM loading in CAS/CCM(20 mg)/FA@Cu-Asp and CAS/CCM(20 mg)@ Cu-Asp

The CCM loading percentages of CAS/CCM(20 mg)@Cu-Asp and CAS/CCM)(20 mg)/FA@Cu-Asp were estimated using a standard calibration curve in water: ethanol (1:1) as the solvent (Fig. S3).

Decomposing of 2.0 mg of CAS/CCM(20 mg)/FA@Cu-Asp (sample 1) and CAS/CCM(20 mg)@Cu-Asp (sample 2) were done in the 2.0 ml mixture of HCl (50 µL, 3.0 M) and ethanol . The absorbance of CCM solutions at 425 nm were 0.613 and 0.669 for samples 1 and 2, respectively, after 5 times dilution. These values were placed in the calibration curve equation, and the corresponding concentrations were obtained. Hence, the CCM concentrations were 51.04 µg ml^-1^ and 55.66 µg ml^-1^, respectively. In other words, 51.04 µg and 55.66 µg of CCM was found in 1000 µg of samples (1) and (2), respectively. The percentage of DLC was 5.10% and 5.57% based on Eq. 1. The total sediment content in samples (1) and (2) was 290.2 and 295.2 mg, respectively. The amount of CCM in the feed was also 20.0 mg. Therefore, the DLE was 74.0% and 82.0% for samples (1) and (2), respectively, based on Eqs. 2.

These values indicated that the acceptable drug loading occurred in both frameworks. According to the previous studies, in situ drug loading during the synthesis process led to more efficient encapsulation of the drug compared to the post encapsulation. In the case of CAS/CCM(20 mg)/FA@Cu-Asp, the presence of FA reduced the CCM loading compared with that of CASS/CCM(20 mg)@Cu-Asp. This can be due to the presence of FA in addition to other biomolecules in the reaction medium that may occupy some of the available functional groups and affect drug loading.

### Investigation of CCM release rate from CAS/CCM(20 mg)@Cu-Asp at different pH and release mechanism

**Fig. 7:**
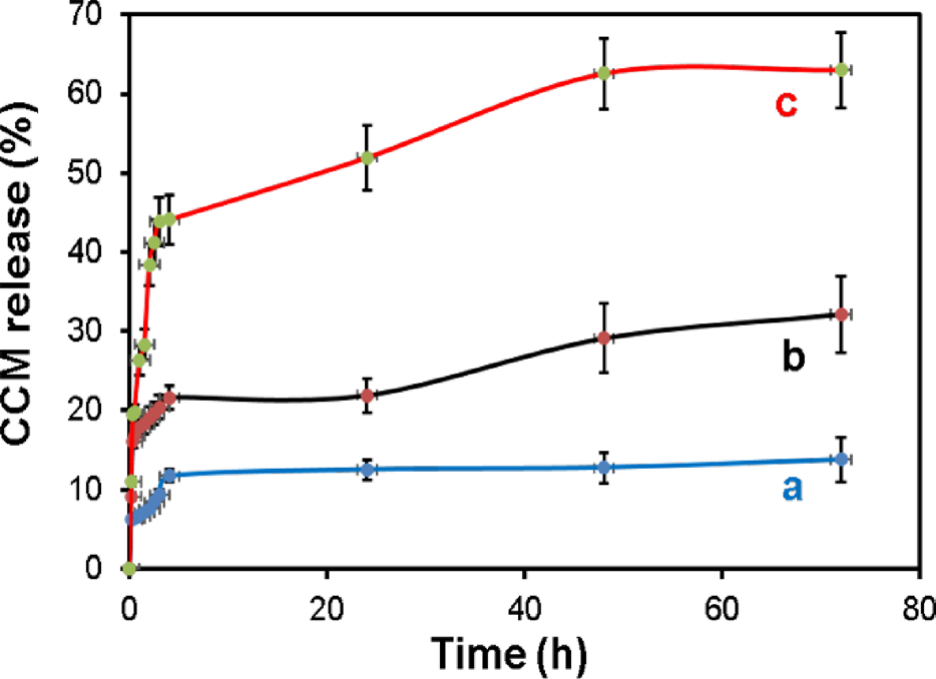
The in vitro CCM release percentage from CAS/CCM (20 mg)@Cu-Asp in different pHs of (**a**) 7.4, (**b**) 6.8 and (**c**) 5.5. Kinetic release data are presented as the average ± SD (n = 3) in three independent experiments.

For the investigation of the release kinetics of CCM from CAS/CCM(20 mg)@Cu-Asp, three different PBS solutions with neutral (pH = 7.4) and acidic pH (pH = 6.8 and 5.5) were selected, which denoted the blood normal condition, secondary endosome, and tumor microenvironment. Figure 7 shows the cumulative release percentage of CCM from CAS/CCM(20 mg)@ Cu-Asp at pHs of 5.5, 6.8, and 7.4 with time.

The release mechanism of CCM from the CAS@Cu-Asp nanocarrier is highly influenced by the pH of the surrounding medium (Fig. 7). The coordination polymer structure formed between Cu^2^⁺ and Asp acted as a pH-responsive matrix, enabling differential drug release profiles at various pH values. At pH 5.5, which mimics the acidic microenvironment of tumor tissues and endosomes, a significantly higher drug release was observed (about 60%). This enhanced release can be attributed to the protonation state of aspartic acid that has two relevant carboxylic acid groups with pK_a_ values around 1.88 and 3.65. At acidic pH, partial protonation of the carboxylate groups of Asp weakend the Cu–aspartate coordination bonds, destabilizing the network and facilitating faster diffusion of CCM. In contrast, at neutral or slightly acidic pH (6.5–7.4), the carboxylate groups remained deprotonated and strongly bound to Cu^2^⁺, resulting in a more stable framework with slower drug release (about 30 and 10%, respectively). This makes the system highly promising for targeted drug delivery in acidic tumor environments.

The drug release data were fitted using non-linear regression to four classical models (Zero-order, First-order, Higuchi, and Korsmeyer–Peppas) (Fig. S6). All R^2^ and parameter values are based on non-linear regression (Table 1). The Korsmeyer–Peppas model showed excellent fitting across all pH conditions for early-time release (Mt/M∞ ≤ 0.6), with high coefficients of determination (R^2^ > 0.93), while the zero-order, first-order, and higuchi models yielded low or even negative R^2^ values, indicating poor overall fit to the full dataset (Table 1).

**Table 1:** Model Parameters and R^2^ Values.

pH	Model	Time range	Parameters	R^2^
7.4	Zero	0–24 h	k = 0.7059	− 2.828
7.4	First	0–24 h	k = 0.0081	− 2.707
7.4	Higuchi	0–2.5 h	k = 6.2458	0.373
7.4	Peppas	0–2.5 h	k = 7.0706, n = 0.1066	0.981
6.8	Zero	0–24 h	k = 1.3346	− 4.466
6.8	First	0–24 h	k = 0.0185	− 4.158
6.8	Higuchi	0–2.5 h	k = 15.3877	0.592
6.8	Peppas	0–2.5 h	k = 17.1591, n = 0.1854	0.941
5.5	Zero	0–24 h	k = 2.9615	− 1.632
5.5	First	0–24 h	k = 0.1982	− 0.094
5.5	Higuchi	0–2.0 h	k = 26.3775	0.96
5.5	Peppas	0–2.0 h	k = 26.6994, n = 0.4258	0.969

The negative R^2^ value indicate a poor fit.

The Korsmeyer–Peppas model is widely applied to investigate the drug release kinetics from polymeric and porous systems. It describes the relationship between the fractional release of drug and time using the empirical equation:

$${\text{M}}_{{\text{t}}} /{\text{M}}_{\infty } = {\text{ k}} \cdot {\text{t}}^{{\text{n}}}$$

where:

M_t_ /M_∞_ is the fraction of drug released at time t, k is a kinetic constant and n is the release exponent indicative of the underlying mechanism (Fickian diffusion (n ≤ 0.45) driven by concentration gradients; Anomalous transport (0.45 < n < 0.89) combining diffusion and matrix relaxation; Case II (n ≥ 0.89) dominated by matrix relaxation or erosion with near zero-order kinetics^62,63^.

Overall, the Korsmeyer–Peppas model best describes the release across all conditions, with n < 0.45 confirming Fickian diffusion. The increase in release rate with decreasing pH is attributed to progressive destabilization of the Cu–Asp framework due to protonation of Asp residues, highlighting its utility as a pH-responsive drug delivery system.

At pH 7.4, the Peppas model yielded k = 7.07 and n = 0.1066 (R^2^ = 0.981), while the Higuchi model fit was relatively poor (k = 6.25, R^2^ = 0.373). At this pH, which is nearly 3.5 units above the pKa of aspartic acid, virtually all of the carboxylate groups on the side chains are deprotonated (> 99.9%), maximizing electrostatic repulsion and thus stabilizing the coordination structure with Cu^2^⁺ ions. The very low n value and poor Higuchi fit reflect slow and purely diffusion-driven release from a dense and rigid framework, with minimal structural degradation or erosion.

At pH 6.8, the Peppas model produced k = 17.16 and n = 0.1854 (R^2^ = 0.941), and the Higuchi model improved slightly (k = 15.39, R^2^ = 0.592). This pH is nearly 2.9 units above the pKa, where carboxylate groups remain largely deprotonated (> 99.5%). However, some weakening of ionic interactions may begin to occur, slightly loosening the coordination matrix. This is evidenced by the increase in both Peppas and Higuchi constants, suggesting enhanced diffusion and a minor contribution from early-stage structural compromise. The n value remains far below 0.45, confirming the dominant Fickian mechanism.

At pH 5.5, the Peppas model gave k = 26.70 and n = 0.4258 (R^2^ = 0.969), and the Higuchi model showed its best fit (k = 26.38, R^2^ = 0.960). At nearly 1.6 units above the pKa, partial protonation of the Asp carboxylate groups (~ 2.5–3%) occurs. This reduction in charge density leads to a destabilization of the Cu–Asp coordination bonds, promoting matrix erosion or partial structural degradation. The excellent Higuchi fit at this pH suggests that diffusion is no longer occurring solely through an intact structure but is accompanied by erosion of the network, which facilitates increased release. The n value approaching 0.45 still supports Fickian diffusion but hints at a transition toward anomalous transport due to matrix compromise.

Taken together, the CAS@Cu–Asp system exhibits pH-responsive release with the Korsmeyer–Peppas model consistently providing the best fit. The Peppas diffusion exponent (n < 0.45) indicates Fickian-controlled release at all pH values. The increase in Higuchi model performance and Peppas constants (k) at lower pH values reflects enhanced diffusion due to structural weakening of the coordination network. These findings highlight the capability of CAS@Cu–Asp nanocarriers to function as intelligent drug delivery systems in acidic microenvironments, such as tumor tissues, where coordination breakdown accelerates therapeutic release.

### Investigation of the toxicity of different Cu-Asp bioMOFs using MTT analysis

**Fig. 8:**
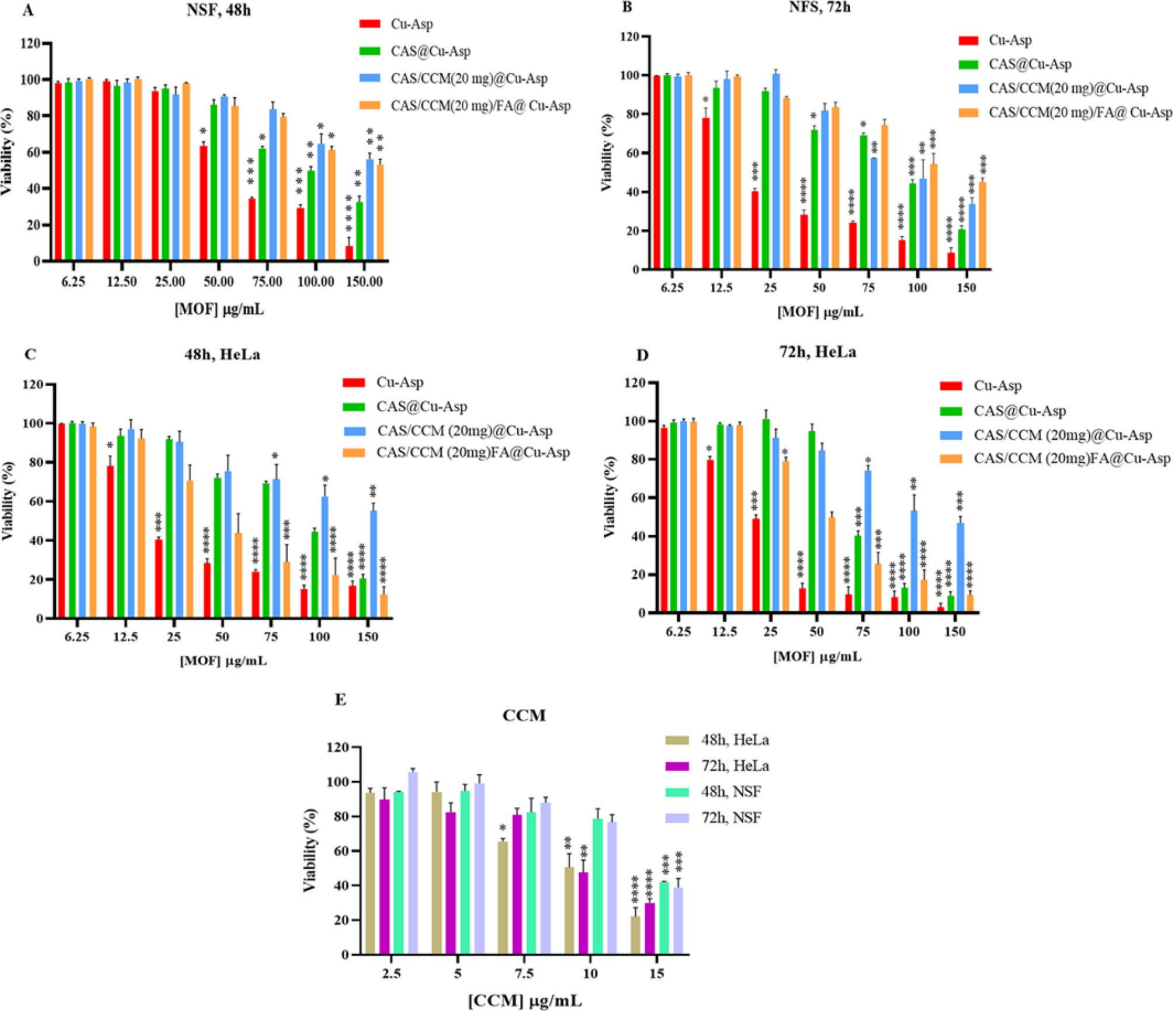
Cell viability (MTT assay) analysis of normal skin fibroblast (NSF) and HeLa cells after treatment with Cu-Asp, CAS@Cu-Asp, CAS/CCM(20 mg)@Cu-Asp, and CAS/CCM(20 mg)/FA@Cu-Asp within 48 h (**A**,**C**) and 72 h (**B**,**D**). MTT assay of NSF and HeLa cells after treatment with CCM (**E**). The statistical significance was determined using the t-test analysis with the following definitions: *(*P* value ≤ 0.05), **(*P* value ≤ 0.01), ***(*P* value ≤ 0.001), and ****(*P* value ≤ 0.0001).

The cytoxicity evaluation of Cu-Asp, CAS@Cu-Asp, CAS/CCM(20 mg)@Cu-Asp, and CAS/CCM(20 mg)/FA@Cu-Asp frameworks was conducted against HeLa cancer cell lines and normal skin fibroblast (NFS) cells using the MTT assay within 48 and 72 h (Fig. 8). SEM images (Fig. 2) revealed that Cu-Asp and CAS@Cu-Asp exhibited rod-shaped morphology, whereas CAS/CCM(20 mg)@Cu-Asp and CAS/CCM(20 mg)/FA@Cu-Asp were nanodots.

As illustrated in Fig. 8A,B, the percentage of cell viability of normal cell fibroblast lines is presented after Cu-Asp treatment for 48 h and 72 h. Within the context of normal skin fibroblasts, a notable toxicity characteristic was observed at a concentration threshold of 50 µg ml^−1^ and exceeding that, following 48 h of Cu-Asp treatment (*P* = 0.034). Accordingly, Cu-Asp functions as a toxic agent due to its cytotoxic effects, starting from a concentration of 50 µg ml^−1^ within 48 h and increasing to 12.5 µg ml^−1^ after 72 h. Interestingly, the introduction of CAS (CAS@Cu-ASP) augmented the toxicity to a level of 75 µg ml^−1^, likely due to interactions between Cu^2^⁺ and CAS ions that inhibited Cu^2^⁺ release during framework degradation. Nevertheless, upon the incorporation of CAS, this statistical significance persisted at the 25 µg ml^−1^, indicating a level of safety for utilization up to this concentration. In addition, after CCM encapsulation in Cu-Asp (CAS/CCM(20 mg) @Cu-Asp), the safety level was increased to 75 and 50 µg ml^−1^ within 48 and 72 h post-treatment, respectively. Moreover, nanocarrier FA functionalization (CAS/CCM(20 mg)/FA@Cu-Asp) retained biosafety up to 75 µgml^-1^at 48 and 72 h, suggesting the potential for administering larger quantities of pharmaceutical agents. Conversely, regarding cell viability against HeLa cancerous cells, significant cell toxicity was noted even at 48 h post-treatment for CAS/CCM(20 mg)/FA@Cu-Asp (25 µg ml^−1^, *P* = 0.041) (Fig. 8C,D). This may be attributed to the innate toxic properties of Cu ions, which are capable of generating reactive oxygen species (ROS), which can be harmful to cancer cells^64^. Indeed, cancer cells do not exhibit resistance to the ROS generated by Cu ions; however, normal cells possess a certain degree of tolerance to this toxicity, enabling them to mitigate the detrimental effects of ROS. In contrast, cancer cells encounter difficulties due to their resistance mechanisms against ROS, a vulnerability that we exploit, implying that our drug delivery system not only administers the drug but also contributes to the induction of toxicity through the nanoparticles themselves^65^. Consequently, in addition to the pharmacological agent (CCM) in framework, Cu ions also induce toxicity, with the distinction that in normal cells, such toxicity becomes evident at a concentration of 75 µgml^-1^, while in cancer cells, it appears that the drug delivery system operates synergistically between the therapeutic agent and the substrate, suggesting that efficacy is not solely attributable to the drug alone. It should be noted that CAS/CCM(20 mg)@Cu-Asp showed biosafety up to 75 μg ml^−1^ on hela cell lines at 48 and 72 h compared to 25 μg ml^−1^ for CAS/CCM(20 mg)/FA@Cu-Asp (Fig. 8C,D). Due to the targeting mechanism employed, the uptake of the drug is significantly enhanced, resulting in a marked increase in toxicity.

Figure 8E shows the results of free CCM toxicity in two normal cell lines and HeLa at different time points. The results show that CCM loading on an ultra-small CAS@Cu-Asp nanocarrier increases its performance relative to that of free CCM. For instance, at a concentration of 75 µg ml^−1^ of the CAS/CCM(20 mg)/FA@Cu-Asp, there is 25.5 µg ml^−1^ CCM. According to Fig. 8E, at a similar concentration, the cell viability percentage of the HeLa cell line at 72 h was 85%, for 25.5 µg ml^−1^ free CCM, whereas the percentage of cell viability for CAS/CCM(20 mg)/FA@Cu-Asp at a concentration of 75 µg ml^−1^ on the HeLa cell line was 25% (Fig. 8E).

Table 2 shows the calculated IC50 values for different Cu-Asp frameworks (Cu-Asp and CAS@Cu-Asp, CASS/CCM(20 mg)@Cu-Asp and CASS/CCM(20 mg)/FA@Cu-Asp) and free CCM in both normal and HeLa cell lines at different time points. Based on these values, the selectivity index (SI), which shows the ratio of IC50 of normal cells to IC50 of cancer cells, was calculated for different samples at 72 h (Table 3). In general, as this number value is greater than 1, the more selective it would be. Therefore, the comparison of the data obtained in Table 3 shows that the calculated SI for the CAS/CCM (20 mg)/FA@ Cu-Asp was higher than CAS/CCM (20 mg)@ Cu-Asp, which indicated the more selectivity of a sample with the FA factor, a result that could be very suitable for treating cancers without causing side effects. Also, the obtained SI for CAS/CCM (20 mg)/FA@ Cu-Asp was greater than free CCM.

**Table 2:** The IC50 values of free CCMs and different Cu-Asp frameworks in HeLa and NSF cell lines within 48 and 72 h of incubation.

		HeLa (µg/ml)	NSF (µg/ml)
CCM	48h	9.884 ± 2.8	13.87 ± 1.71
	72h	10.62 ± 2.6	13.38 ± 2.12
Cu-Asp	48h	51.08 ± 1.19	56.35 ± 1.72
	72h	13.64 ± 1.56	12.95 ± 1.81
CAS@Cu-Asp	48h	82.22 ± 1.70	87.37 ± 1.10
	72h	53.08 ± 1.22	48.79 ± 1.45
CAS/CCM (20 mg)@Cu-Asp	48h	> 100	> 100
	72h	> 100	90.13 ± 2.2
CAS/CCM (20 mg)/FA@Cu-Asp	48h	44.26 ± 3.0	> 100
	72h	46.63 ± 1.6	> 100

**Table 3:** Calculation of the selectivity index (SI) for different samples after 72 h.

Sample	SI = IC50Normal/ IC50HeLa
Free CCM	1.26
Cu-Asp	0.95
CAS@Cu-Asp	0.90
CAS/CCM(20mg)@Cu-Asp	0.90
CAS/CCM(20mg)/FA@Cu-Asp	2.15

Table 4 provides a comparative overview of various MOF-based systems used for CCM delivery, demonstrating that the present formulation exhibits competitive or superior performance in terms of drug loading, pH-responsive release, and SI value.

**Table 4:** A comparative overview of various MOF-based systems used for CCM delivery.

Carriers for cancer treatment	Loading method	DLC	DLE	SI	IC50 values of CCM@MOF in cancer cell lines	pH-responsive release	References
^1^CCM-ZIF-8	In situ CCM loading	3.42%	83.33%	–	–	pH 5.5 = 88% pH 7.4 = 28% after 7 h	^66^
GA/CCM/FA@ZIF-8	In situ CCM loading and post loading	3.3%	90%	2.70	20.2 μg/mL	pH 5.5 = 72% pH 7.4 = 43% after 24 h	^44^
CCM@ZIF-8	In CCM loading	12.93	85.91	–	515.86 μg/mL	pH 5.5 = 68.47% pH 7.4 = 55% after 120 h	^67^
^2^Gelatin@ β-CD MOF@CCM	Post CCM loading	55.63%	83.45%		–	pH 5.5 = 55.98% pH 7.4 = 9.24% after 72 h	^68^
^3^DMOF-1 and DMOF-1-NO_2_	Post CCM loading	22.4 and 28.3%	–	–	–	pH 5.5 = 55.98% pH 7.4 = 9.24% after 5 h	^69^
^4^IRMOF-3@CCM@FA	In situ CCM loading	52%	98%	–	45.0 µM	pH 5.5 = 55% pH 7.4 = 31% after 24 h	^70^
^5^MIL-101(Fe)@CCM	Post CCM loading	56.3%	48.7%	–	72.6 μg/mL	pH 5.5 = 64.7% pH 7.4 = 26% after 22 days	^71^
^6^CCM@N_3_-bio-MOF-100/FA	Post CCM loading	–	25.64%	–	15.34 μM	pH 5.5 = 87.2% pH 7.4 = 77.4%	^72^
CAS/CCM(20 mg)/FA@Cu-Asp	In situ CCM loading	5.10%	74%	3.2	46.63 µg/ml	pH 5.5 = 65% pH 7.4 = 25% after 72 h	This study

^1^ZIF-8: Zn^2+^ and 2-methyl imidazole.

^2^β-CD MOF: K^+^ and β-Cyclodextrine.

^3^MIL-101: Fe^3+^ and terephthalic acid.

^4^DMOF-1: {Zn(BDC)(DABCO)_0.5_}_n_, (BDC^2-^: 1,4-benzene dicarboxylate, DABCO: 1,4-Diazabicyclo[2.2.2]octane).

^5^IRMOF: Zn^2^⁺ and NH_2_-H_2_BDC.

^6^N_3_-bio-MOF-100: Zn^2+^ and 2-azidobiphenyldicarboxylic acid (N3-BPDC).

## Conclusion

This study presents the development of an ultra-small nanocarries using copper-aspartate (Cu-Asp) bioMOFs for targeted CCM delivery. A novel one-pot method was employed to encapsulate the anticancer drug CCM within the CAS@Cu-Asp BionanoMOF. CAS significantly enhanced the biocompatibility of Cu-Asp. The formation of the CAS/CCM complex can serve as a template for the biomineralization process using bioMOF. Different amounts of CCM (10, 15, and 20 mg) were loaded onto the CAS@Cu-Asp system. An increase in the CCM amount altered the Cu-Asp morphology from rod-shaped to ultra-small nanodots. Additionally, the poor water solubility and rapid degradation of CCM under physiological conditions were addressed by incorporating it into the CAS/CCM@Cu-Asp nanocarrier. This enhancement is attributed to the interactions between CCM and the functional groups of CAS, as well as the complexation between CCM and Cu^2^⁺ ions. The targeting of the CAS/CCM@Cu-Asp nanocarrier was achieved via FA in an alkaline Asp medium at room temperature, without the use of toxic, excessive organic solvents or additional conjugation agents. The CCM release from CAS/CCM@Cu-Asp framework followed a pH-responsive Fickian diffusion mechanism, best described by the Peppas model. At lower pH, partial protonation of aspartate weakened the coordination structure, increasing release rates. These results highlight the potential of Cu–Asp systems for controlled drug delivery in acidic environments. Furthermore, the one-pot method resulted in a high CCM loading. The MTT assay results on two different cell lines, NSF and Hela, demonstrated that CAS/CCM(20 mg)/FA@Cu-Asp nanocarrier showed promising potential for targeted treatment of Hela cancer cells, with an SI of 2.15.

## Supplementary Information

Below is the link to the electronic supplementary material.

Supplementary Material 1

## Data Availability

All data generated or analyzed during this study are included in the published article and its supplementary information files.
