# Supplementary material for: Bioinspired synthesis of ultra-small copper-aspartate BioMOF nanodots using sodium caseinate for targeted curcumin delivery
Source: Sci Rep. 2025 Oct 8;15:35086. doi: 10.1038/s41598-025-18880-4 (PMC12508201; doi:10.1038/s41598-025-18880-4)
Supplement: Supplementary file 1 — Supplementary Material 1 [file 41598_2025_18880_MOESM1_ESM.docx]

**Bioinspired Synthesis of Ultra-Small Copper-Aspartate BioMOF Nanodots using Sodium Caseinate for Targeted Curcumin Delivery**

Reyhane Rezaee^a^, Maryam Tohidi*^a^, Banafsheh Rastegari*^b^, and Sedigheh Zeinali^a^

^d^Department of Nanochemical Engineering, Faculty of Advanced Technologies, Shiraz University, Shiraz, Iran, postal code: 71946-84334. *E-mail: matohodi@Shirazu.ac.ir

^b^Diagnostic Laboratory Sciences and Technology Research Center, School of Paramedical Sciences, Shiraz University of Medical Sciences, Shiraz, Iran, Postal code: 71837-53335 *E-mail: brastegari@sums.ac.ir

Fig. S1. CAS@Cu-Asp synthesis steps at room temperature: a) alkaline solution of CAS/Asp, b) aqueous solution of Cu^2+^, C) addition of Cu^2+^ to the CAS/Asp solution and homogenization, d) centrifuged sample, and e) final product (CAS@Cu-Asp).


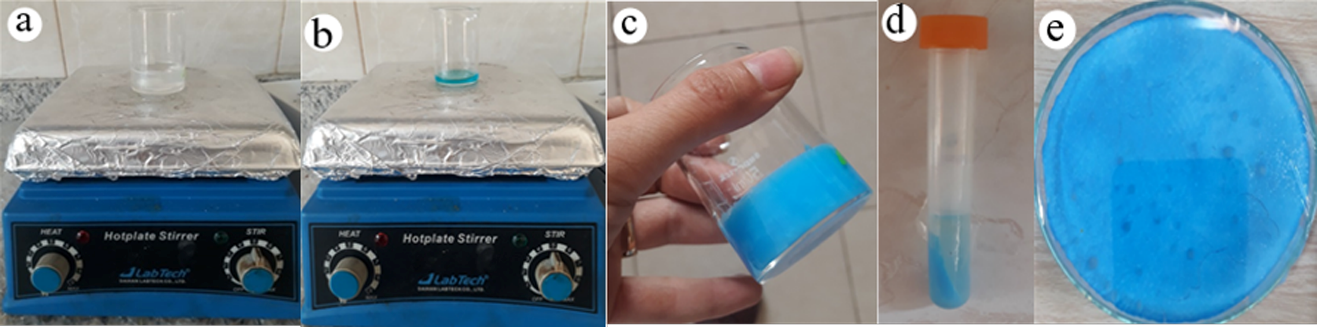

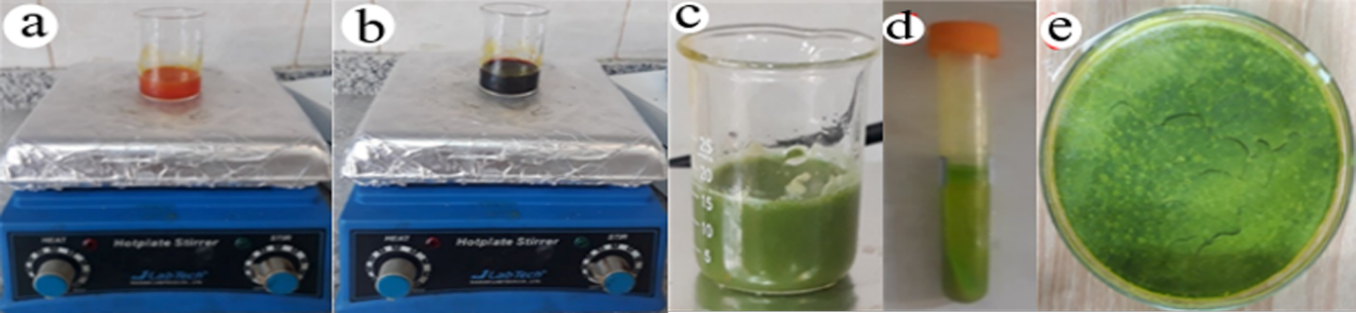


Fig S2. CAS/CCM@Cu-Asp synthesis steps at room temperature: a) CCM/CAS mixture, b) addition of the alkaline Asp solution to the CCM/CAS mixture, c) addition of Cu^2+^ solution to the CCM/CAS/Asp mixture and homogenization, d) centrifuged sample, and e) final product (CAS/CCM@Cu-Asp).


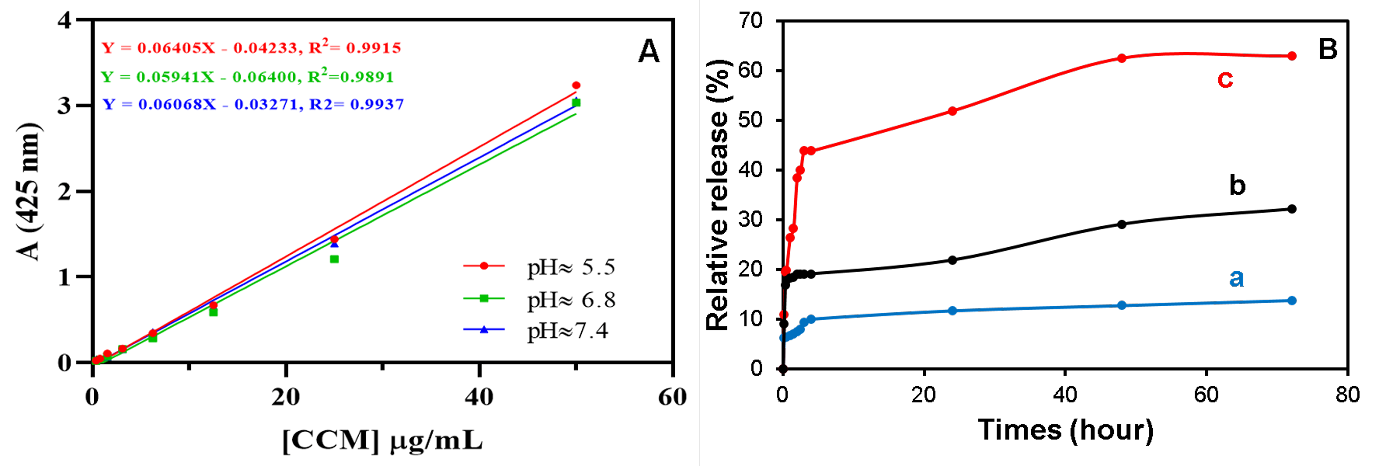


Figure S3. Standard curves of CCM at different pH values including pH$\sim$5.5, 6.8, and 7.4.


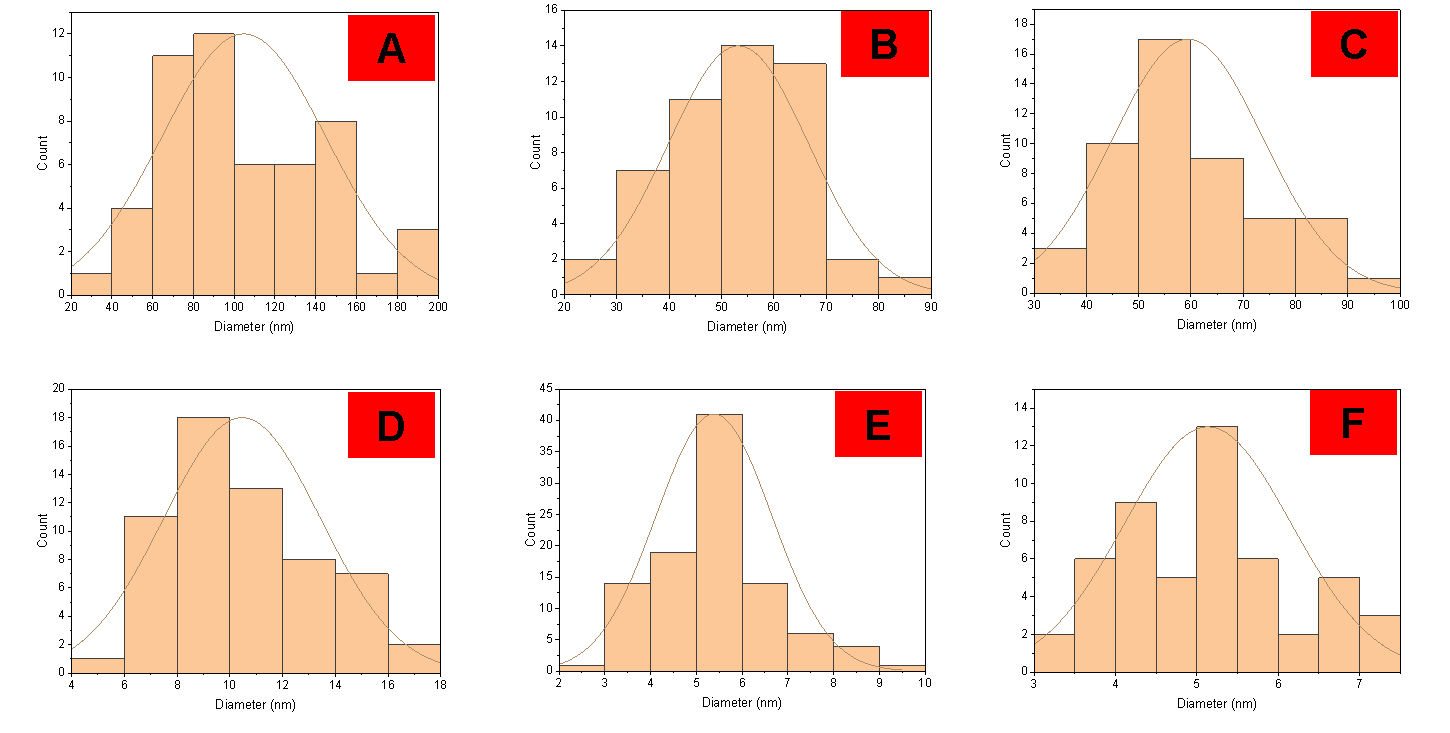


Fig. S4. Size distribution histogram of A) Cu-Asp, B) CAS@Cu-Asp, C) CAS/CCM (10 mg)@Cu-Asp, D) CAS/CCM (15 mg)@Cu-Asp, E) CAS/CCM (20 mg)@Cu-Asp and F) CAS/CCM (20 mg)/FA@Cu-Asp.


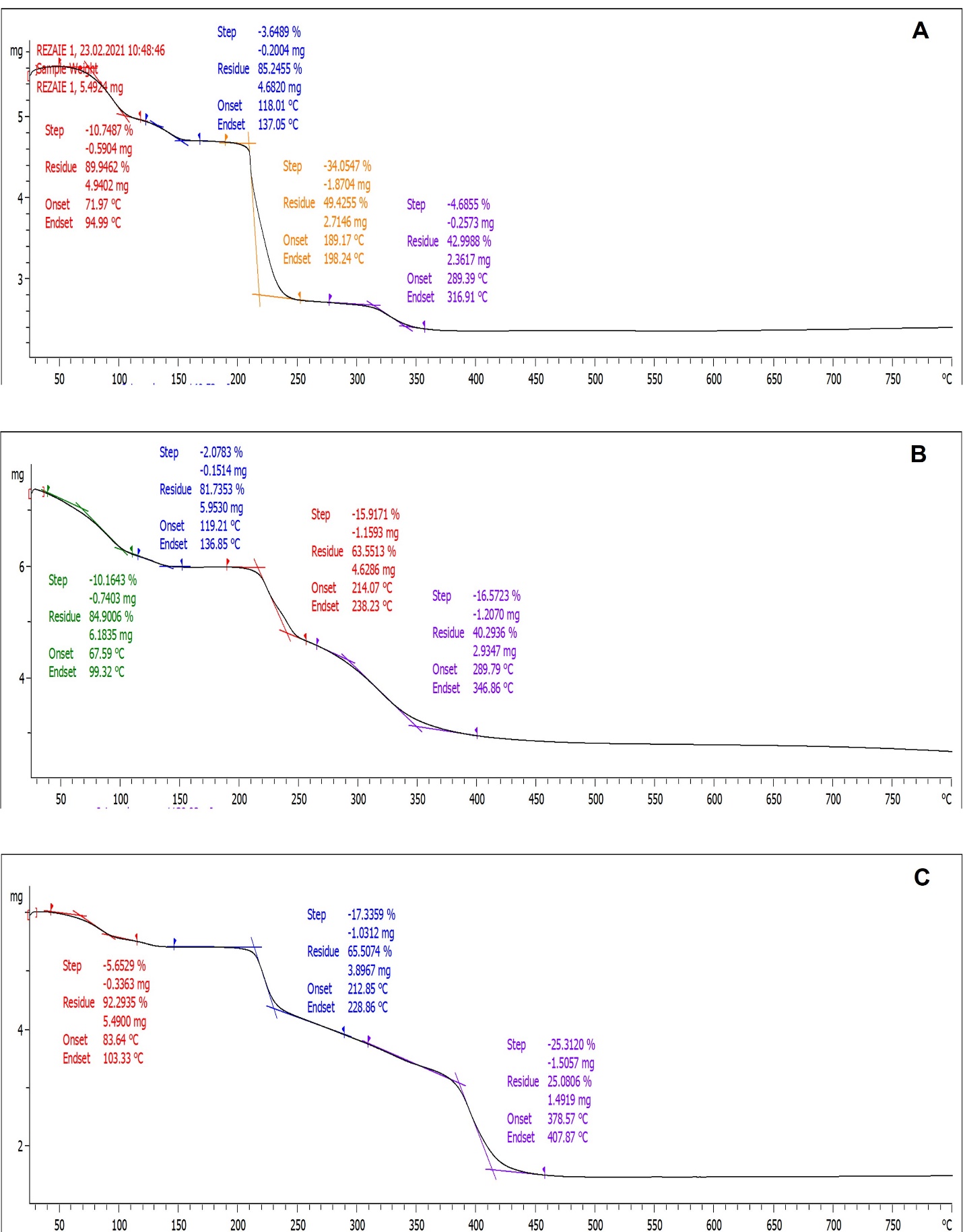


Fig. S5. TGA thermograms of A) Cu-CAS, B) CAS@Cu-Asp and C) CAS/CCM (20 mg)/FA@Cu-Asp.


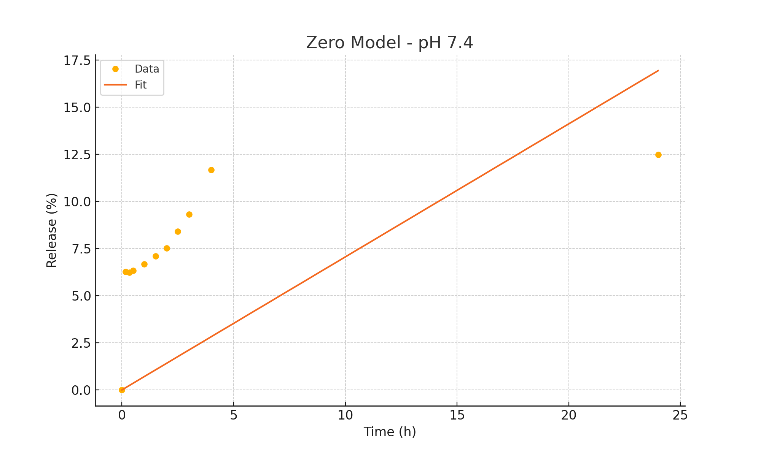

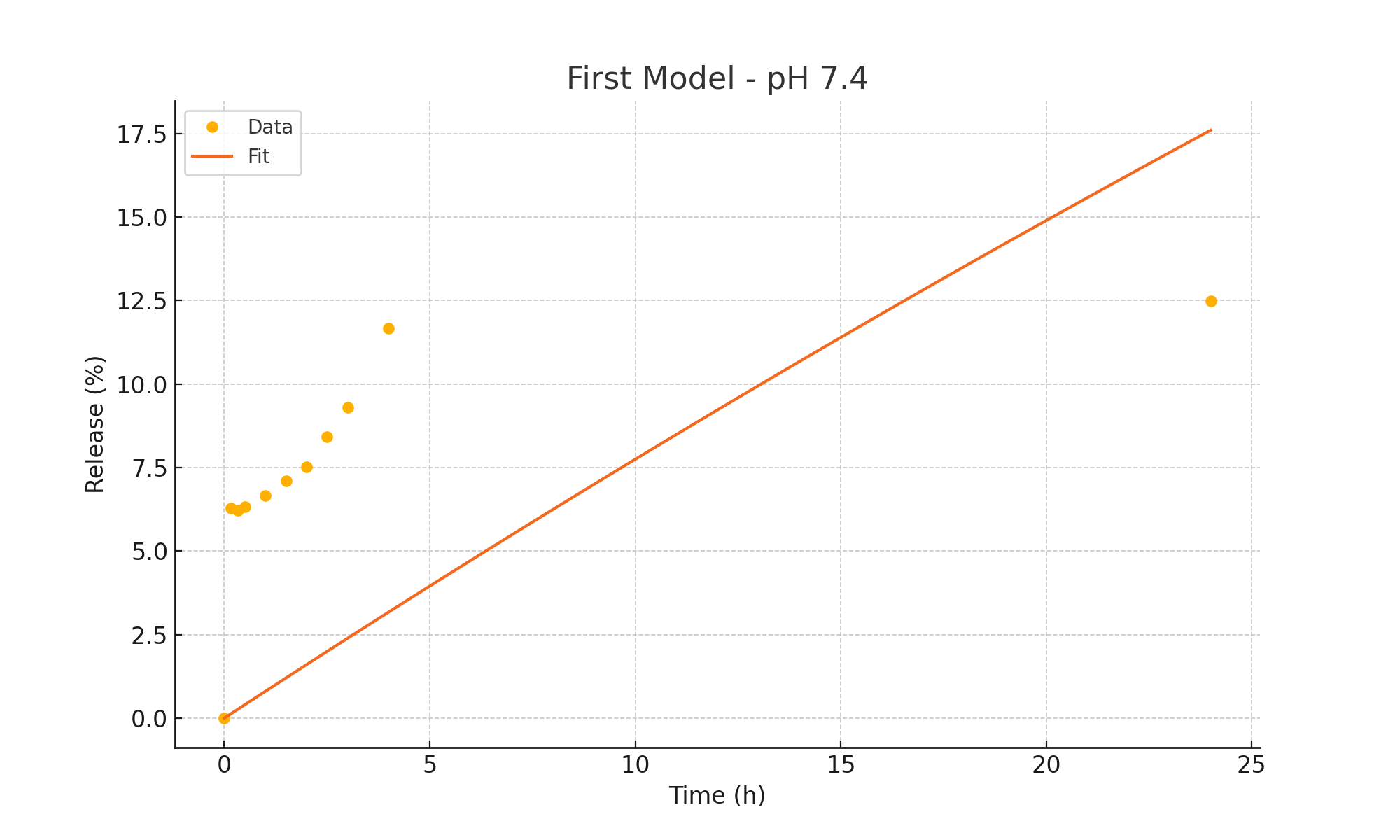

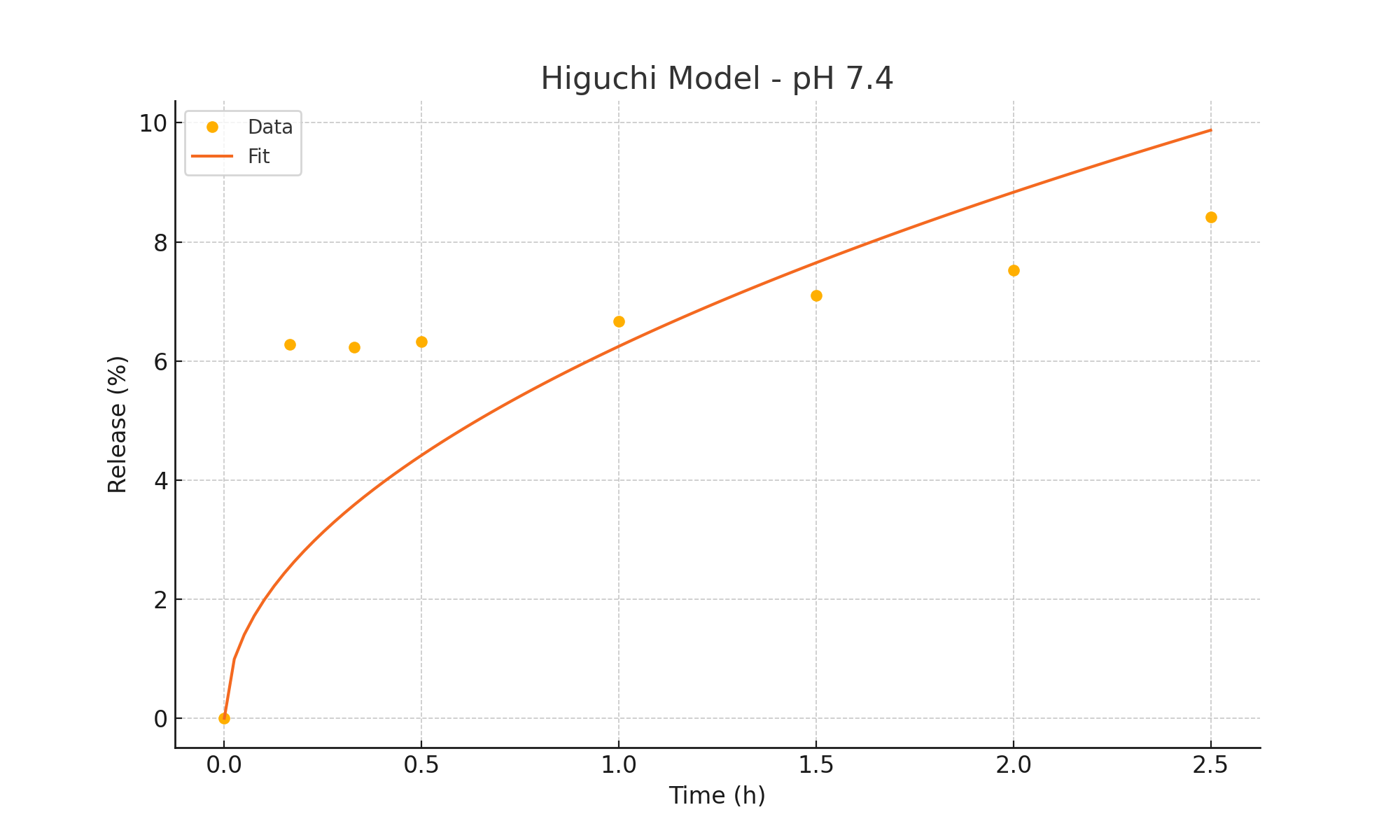

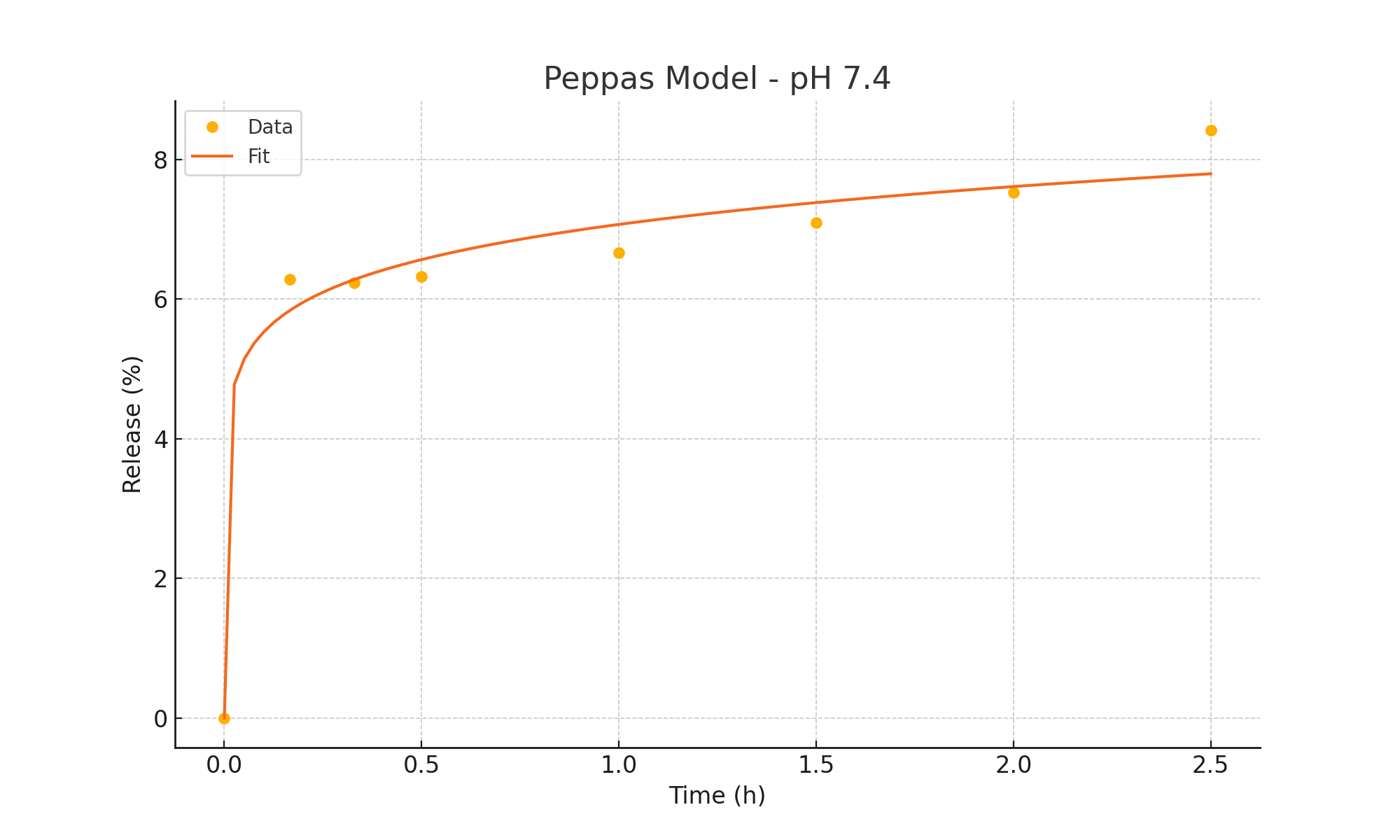

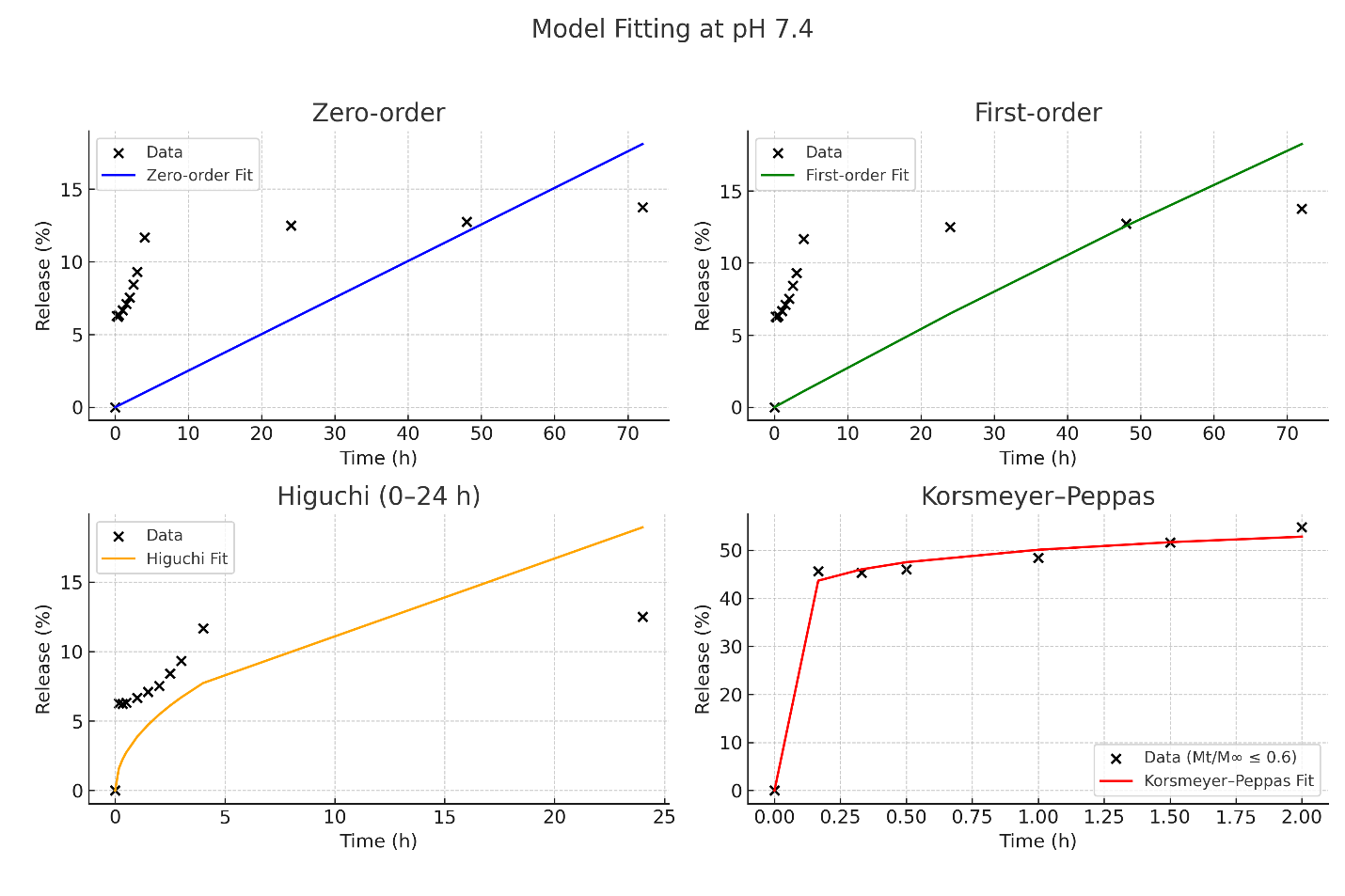

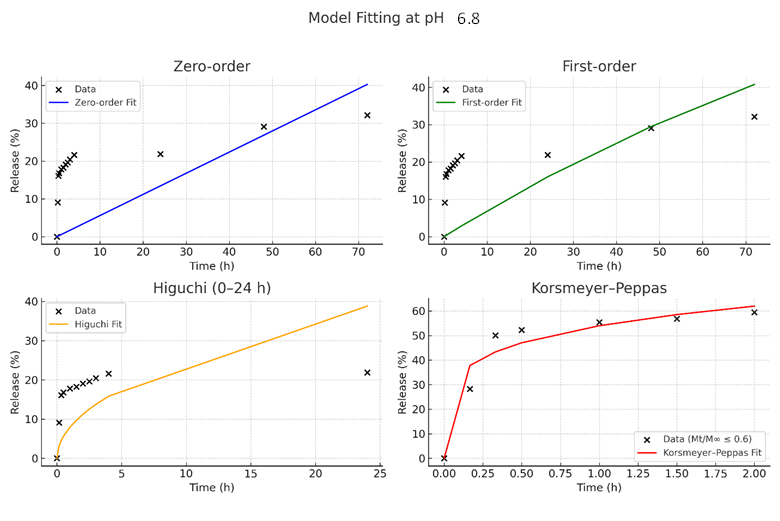

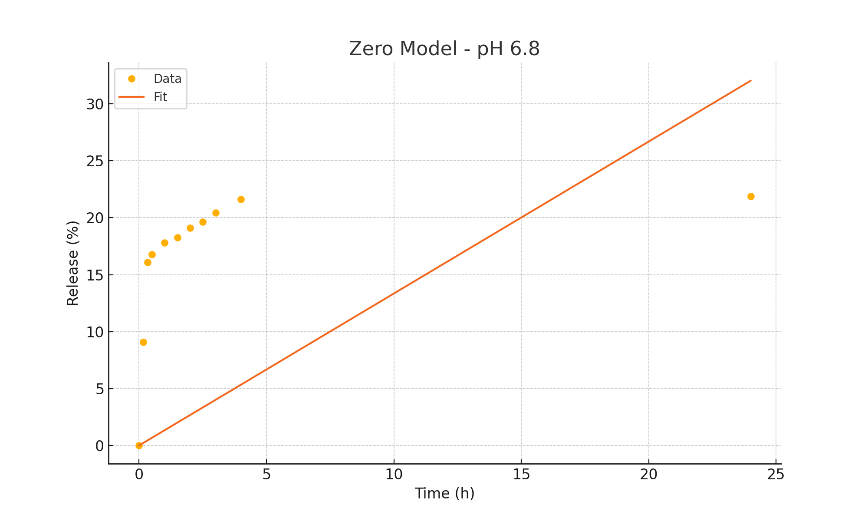

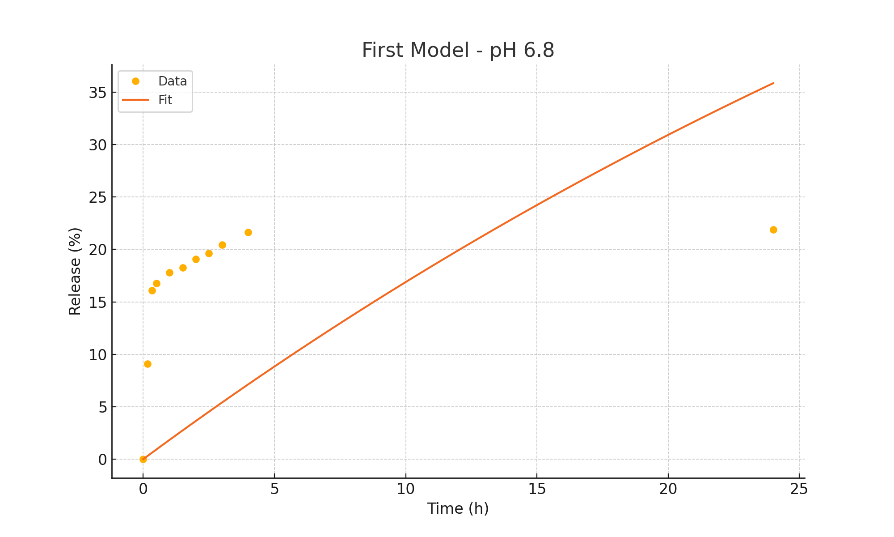

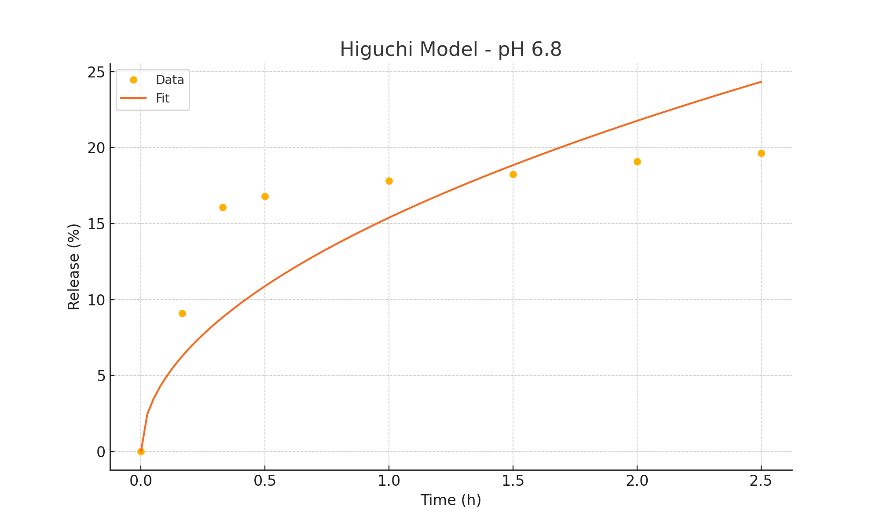

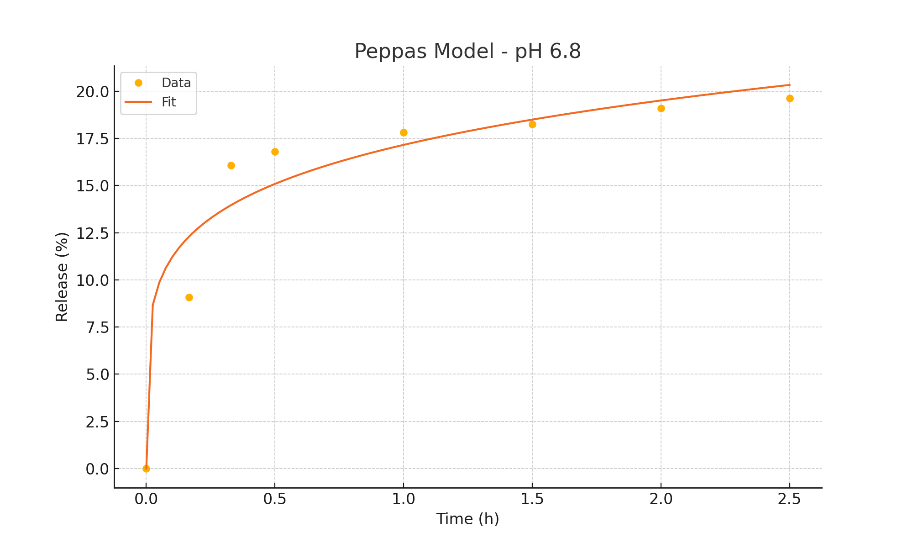

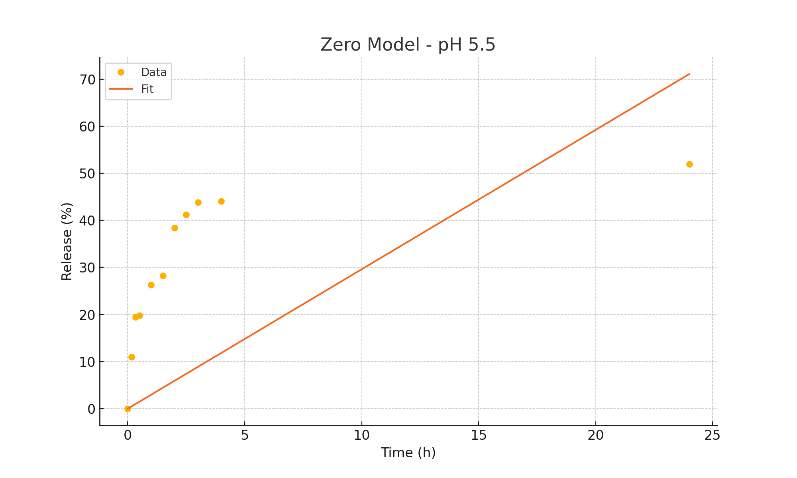

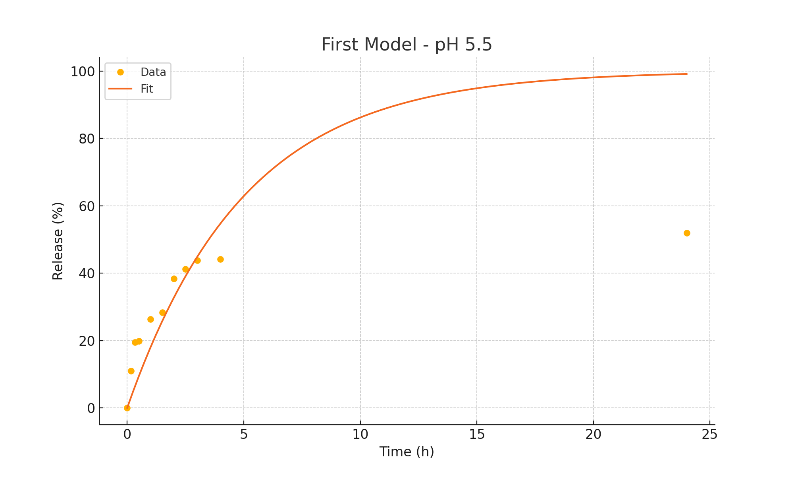

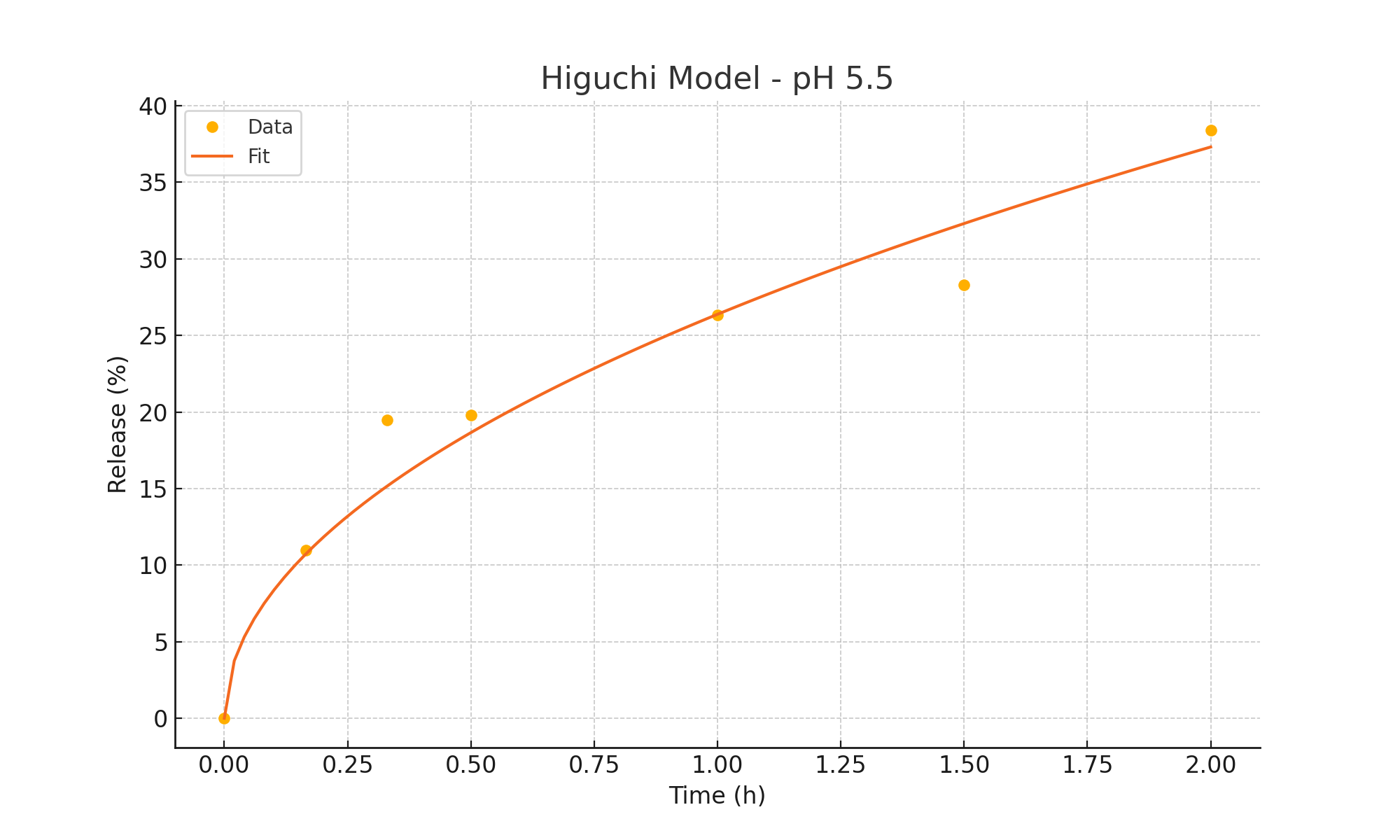

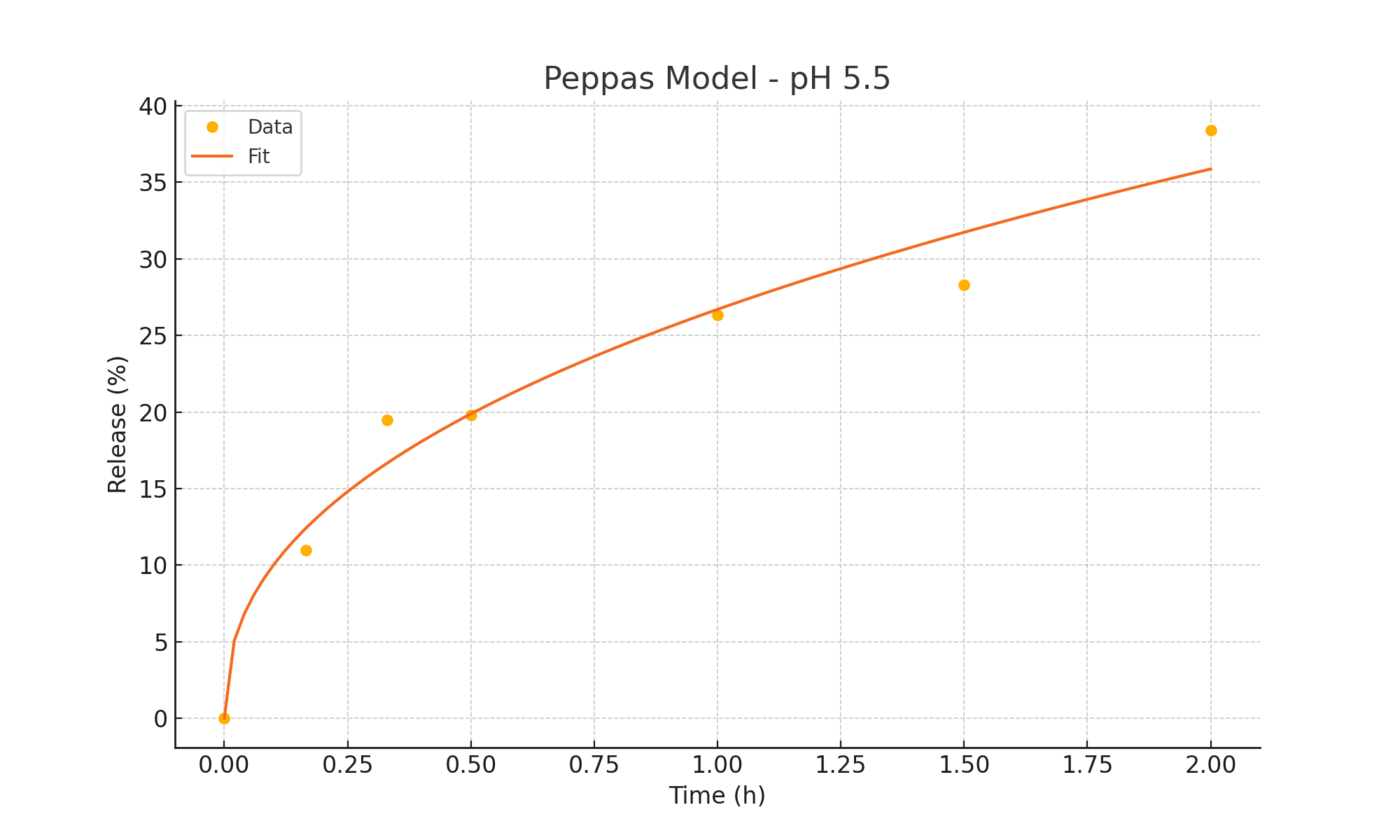

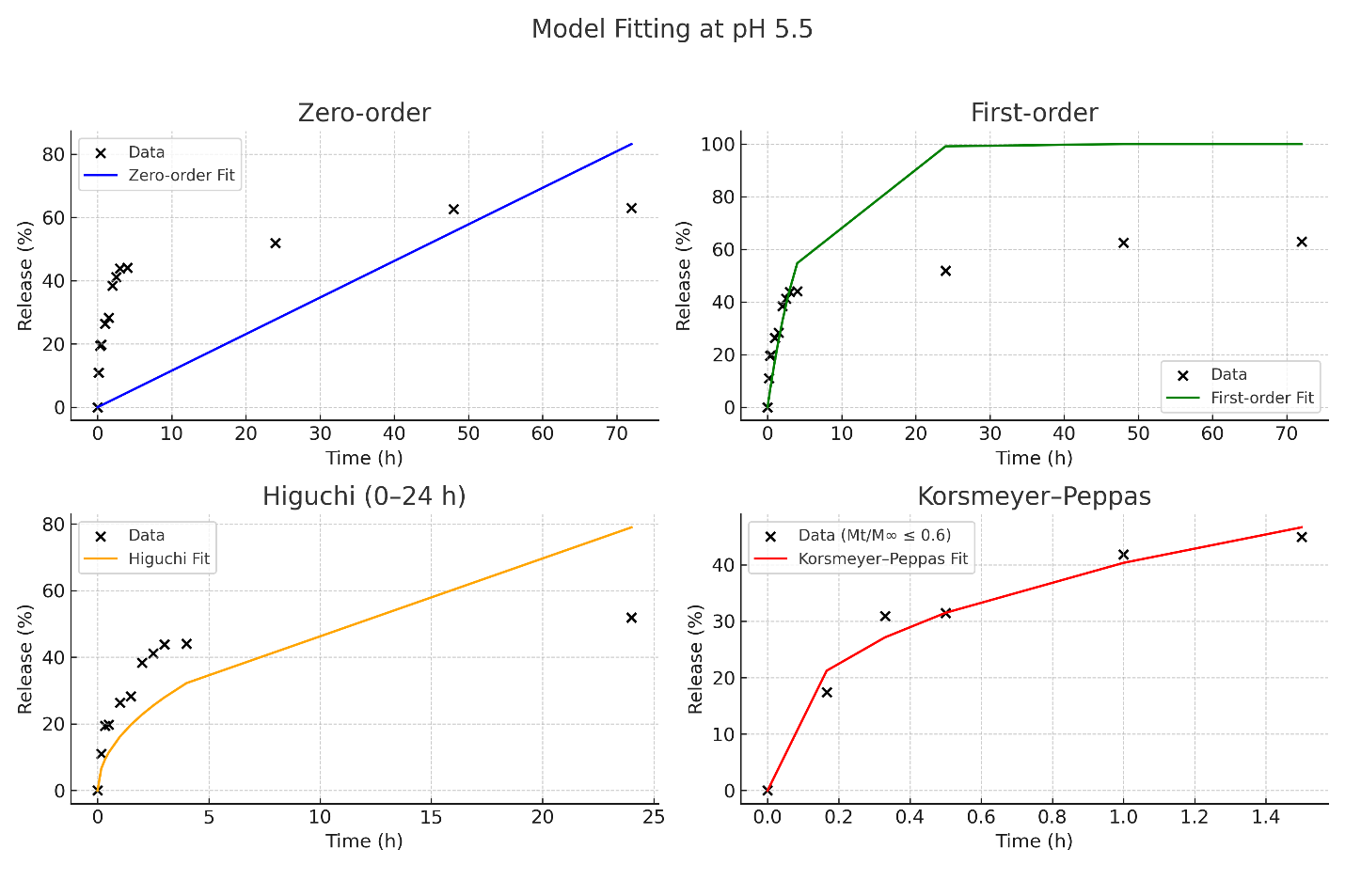


Fig. S6. Fitted drug release profiles using non-linear regression to the four classical models (Zero-order, First-order, Higuchi and Korsmeyer–Peppas models at pH 7.4, 6.5, and 5.5.
